# D-Penicillamine/Dihydroquercetin Dual-Loaded Metal–Organic Framework as a Microenvironment Copper Regulator for Enhancing the Therapeutic Efficacy of Polyphenolic Antioxidant in Alzheimer’s Disease

**DOI:** 10.3390/molecules31010111

**Published:** 2025-12-28

**Authors:** Xuhan Wu, Gang Huang, Licong Chen, Yiling Xie, Qi Ding, Enpeng Xi, Yun Zhao, Nan Gao

**Affiliations:** Key Laboratory of Polyoxometalate and Reticular Material Chemistry of Ministry of Education, Faculty of Chemistry, Northeast Normal University, Changchun 130024, China

**Keywords:** Alzheimer’s disease, polyphenol compounds, dihydroquercetin, D-penicillamine, metal microenvironment regulator

## Abstract

Polyphenols like dihydroquercetin, rutin, and rifampicin show promise for Alzheimer’s disease (AD) therapy due to their ability to inhibit amyloid-β (Aβ) aggregation and reduce reactive oxygen species (ROS), garnering significant recent interest. However, their efficacy is substantially diminished because excess metal ions present in amyloid plaques can chelate these compounds. Therefore, reshaping the metal microenvironment in the patient’s brain is particularly important for the therapeutic effect of AD. To address the above issues, we have constructed a composite system formed by NH_2_-MIL-101(Fe) (MOF), dihydroquercetin (DHQ), and D-penicillamine (D-pen). Due to the lack of π-π interaction and the low adsorption energy between D-pen/MOF, the release order and speed of D-pen was much faster than DHQ, thus achieving metal microenvironment regulation and ensuring the therapeutic effect of DHQ. In a 5 × FAD transgenic mouse model, DD@MOF treated and improved spatial learning and memory deficits. Therefore, the DD@MOF based on polyphenolic compounds provides a potential research direction for intervention in Alzheimer’s disease through chelating copper ions and antioxidant properties.

## 1. Introduction

Alzheimer’s disease (AD), representing the predominant type of dementia globally, imposes significant socioeconomic burdens with its rising prevalence among aging populations. While the precise etiology remains incompletely elucidated, neuropathological hallmarks prominently feature extracellular amyloid-β (Aβ) plaque deposition and elevated oxidative stress markers [1,2,3,4]. These pathological signatures have driven research into therapeutic agents capable of dual-pathway intervention. Notably, certain polyphenolic structures—including dihydroquercetin, rutin, and rifampicin derivatives—have emerged as promising candidates due to their demonstrated capacity to simultaneously disrupt Aβ fibrillization and neutralize reactive oxygen species (ROS), positioning them at the forefront of recent AD therapeutic exploration [5,6,7,8]. However, previous studies indicated that metal ions were involved in Aβ aggregation and neurotoxicity. Significant high levels of metal ions, such as Cu ≈ 0.4 × 10^−3^ M, were found in the amyloid plaques of the brain tissue in patients with Alzheimer’s disease [9,10,11]. These metal ions can be chelated by polyphenol compounds; in this way, the therapeutic effects of polyphenolic compounds have been weakened, which urgently needs to be addressed in the treatment of AD. Therefore, a kind of metal microenvironment regulator was needed to improve the therapeutic effect of polyphenolic compounds in AD.

**Scheme 1:**
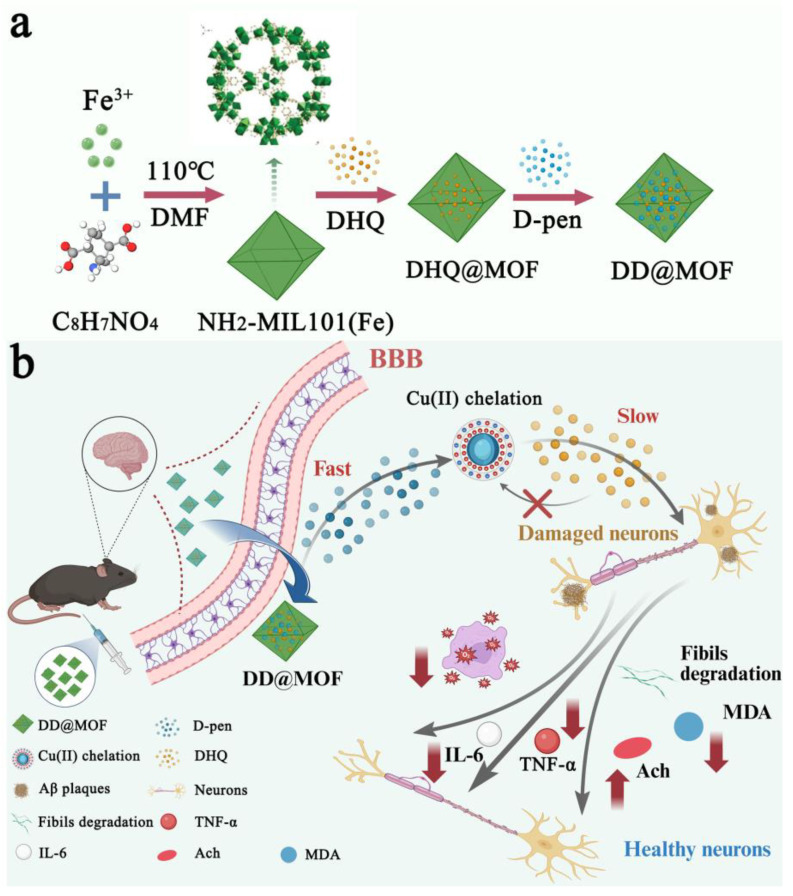
Synthesis of DD@MOF and Its Synergistic Therapeutic Mechanism in an Alzheimer’s Disease Model. Figure (**a**): Synthesis pathway of DD@MOF nanoparticles. Figure (**b**): illustrates the in vivo mechanism of DD@MOF in an Alzheimer’s disease mouse model. After intraperitoneal injection, the nanoparticles can rapidly cross the blood-brain barrier and enter brain tissue. Within the brain, they exert multiple synergistic therapeutic effects.

More importantly, there must be a strict release sequence between the metal microenvironment regulator and polyphenolic compounds, where the metal microenvironment regulator could be released first to achieve the pre-clearance of metal ions. To investigate carrier–drug interactions that critically govern payload dynamics, we developed a sequential delivery platform based on NH_2_-MIL-101(Fe), an iron-based MOF synthesized from 2-aminoterephthalic acid (AAPD) and Fe^3+^ [12,13,14,15,16,17], and depending on the intermolecular interactions, the dihydroquercetin (DHQ, a well-known anti-AD polyphenolic compound) was loaded into the MOF first. Then, the D (-)-Penicillamine (D-pen, a heavy metal ion detoxifier) was also loaded in the MOF [16,18,19,20,21,22,23,24,25]. Due to the lack of π-π interaction and the low adsorption energy between D-pen/AAPD, the release order and speed of D-pen was much faster than DHQ, thus achieving metal microenvironment regulation and ensuring the therapeutic effect of DHQ. The neurodegenerative pathology of Alzheimer’s disease (AD) necessitates precise drug delivery systems capable of traversing the blood–brain barrier (BBB) and achieving controlled release within deep brain tissues. NH_2_-MIL-101(Fe)(MOF) with a size < 200 nm can be obtained by microwave-assisted hydrothermal synthesis, so it can penetrate the BBB [26]. In the 5 × FAD transgenic mouse model, the study observed that DD@MOF treatment reduced the accumulation of metal ions and Aβ plaques in the brain and improved spatial learning and memory function. These findings provide preliminary evidence for an AD therapeutic strategy based on polyphenol compounds (Scheme 1).

## 2. Results

### 2.1. Synthesis and Characterization of MOF

Firstly, MOF was successfully synthesized using FeCl_3_·6H_2_O and AAPD as raw materials using a microblog-assisted method. The final crystals obtained can be used for subsequent drug loading [27,28].

**Figure 1:**
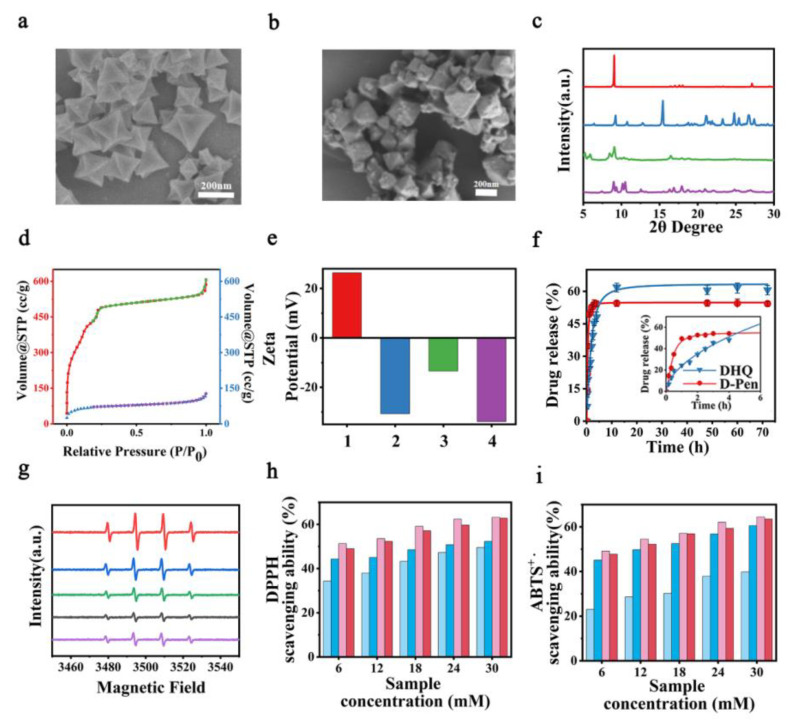
Structural characterization NH_2_-MIL-101(Fe) and DD@MOF. (**a**) SEM image of MOF. (**b**) SEM image of DD@MOF. (**c**) XRD images. (**d**) N_2_ adsorption–desorption isotherms of MOF and DD@MOF (red: adsorption of MOF; green: desorption of MOF; blue: adsorption of DD@MOF; purple: desorption of DD@MOF). (**e**) Zata potential of 1, 2, 3, and 4. (1: MOF; 2: DHQ@MOF; 3: D-pen@MOF; 4: DD@MOF). (**f**) Release process of DHQ and D-pen from DD@MOF for 72 h (inset: release process of DHQ and D-pen from DD@MOF for 6 h). (**g**) The antioxidant capacity of different substances was tested by EPR. (red: free; blue: Cu^2+^ + DHQ; green: Cu^2+^ + DHQ@MOF; black: DD@MOF; purple: Cu^2+^ + DD@MOF). (**h**) Determination of antioxidant capacity of different concentrations of DD@MOF and corresponding contents of Cu^2+^ + DHQ, Cu^2+^ + DHQ@MOF, DD@MOF, and Cu^2+^ + DD@MOF by DPPH method (light blue: Cu^2+^ + DHQ; deep blue: Cu^2+^ + DHQ@MOF; pink: DD@MOF; red: Cu^2+^ + DD@MOF). (**i**) Determination of antioxidant capacity of different concentrations of DR@MOF and corresponding contents of MOF, DHQ, and D-pen by ABTS method (light blue: Cu^2+^ + DHQ; deep blue: Cu^2+^ + DHQ@MOF; pink: DD@MOF; red: Cu^2+^ + DD@MOF). NH_2_-MIL-101(Fe) is uniformly referred to as MOF in the image.

The morphological and structural properties of the synthesized materials were systematically characterized. SEM analysis revealed that both NH_2_-MIL-101(Fe) and drug-loaded DD@MOF maintained well-defined octahedral morphology with smooth surfaces, showing no significant particle size variation after DHQ and D-pen loading (Figure 1a,b). This can be further proved by the TEM image (Figure S1, Supplementary Materials). Similarly, DLS confirmed the size of DD@MOF to be around 200 nm, which is consistent with the TEM results, indicating that the DD@MOF are suitable for cellular and biological applications (Figure S1, Supplementary Materials). The synthesized crystals were examined using X-ray diffraction (XRD). The XRD diffraction diagram obtained were consistent with simulation, and the characteristic diffraction peaks ≈ 2θ = 9° and 2θ = 11° were able to coincide, confirming the successful synthesis of MOF. After loading DHQ and D-pen, DD @ MOF still had the characteristic peaks of MOF and no obvious drug characteristic peaks, which confirmed that the drug molecules were successfully encapsulated in the internal pores of MOF. According to the results of XRD, we can infer that both NH_2_-MIL-101(Fe) and DD@MOF were successfully synthesized (Figure 1c).

The N_2_ adsorption–desorption isotherms show that MIL-101(Fe)-NH_2_ exhibits a typical Type I isotherm with an H4 hysteresis loop, indicating a hierarchical pore structure dominated by micropores along with some mesopores. Its BET specific surface area is as high as 1520 m^2^·g^−1^. After successful loading of the drugs D-pen and DHQ to form DD@MOF, the specific surface area of the material sharply decreases to 232 m^2^·g^−1^. This nearly 85% reduction in specific surface area serves as direct and strong evidence that the drug molecules have successfully entered and occupied the pore channels of the MOF. It clearly rules out the possibility that the drugs are merely physically adsorbed on the external surface of the material, as physical adsorption typically does not lead to such significant pore filling (Figure 1d, Figures S3 and S4, Supplementary Materials). Zeta potential analysis further confirms the successful synthesis of DD@MOF. MIL-101(Fe)-NH_2_ exhibits a positive potential of +28.36 mV in an aqueous dispersion system, which is primarily attributed to the protonation of its abundant surface amino (-NH_2_) groups under the test conditions, resulting in a positive charge. Upon successful loading of D-pen and DHQ, the resulting DD@MOF shows a sharp reversal in Zeta potential to a negative value of −31.29 mV. This fundamental shift from a significantly positive to a significantly negative potential serves as direct evidence that the drug molecules have been successfully grafted onto the MOF framework through chemical interactions (Figure 1e). Infrared spectroscopy analysis revealed that in the DD@MOF spectrum, the C=O peak of DHQ (1627 cm^−1^), the N-H peaks of D-Pen (3183 cm^−1^), and the characteristic S-H peak (2512 cm^−1^) were observed. Simultaneously, the broad -NH_2_ peak of MOF (3346 cm^−1^) exhibited a red shift, indicating the successful loading of D-Pen and DHQ onto NH2-MIL-101(Fe) (Figure S5, Supplementary Materials). In addition, the presence of DHQ and D-pen in MOF was also confirmed by thermal gravimetric analysis spectroscopic measurements, which further justified the conclusion drawn from our experiments (Figure S6, Supplementary Materials). Subsequent stability assessments demonstrated that DD@MOF maintained structural integrity in PBS over 7 days, as evidenced by preserved crystallinity in XRD patterns and consistent hydrodynamic diameter in DLS measurements (Figures S7 and S8, Supplementary Materials). This further proves that DD@MOF has excellent stability and will not release metal ions in the body.

### 2.2. Loading the Drug in MOF and Drug Release

The maximum UV absorption wavelength of DHQ was selected as λ = 291 nm (Figure S9, Supplementary Materials). The standard curve of DHQ in H_2_O was drawn by using the different absorbance values of DHQ in H_2_O with different concentrations (Figure S10, Supplementary Materials). In order to select the most suitable DLC and DLE, we incubated MOF and DHQ with different mass ratios for 24 h and tested their drug loading capacity (DLC) and drug loading efficiency (DLE), respectively (Table S1, Supplementary Materials). Through the change of the mass ratio of MOF/DHQ, we can conclude that with the increase of DHQ mass, the DLC value decreases first and then increases, and the DLE value increases first and then decreases. Several studies have shown that the interaction between the metal sites in NH_2_-MIL-101(Fe) and the hydroxyl group of DHQ provided drug encapsulation with high DLC. The release of drugs with different mass ratios in PBS with pH = 7.4 was further investigated, and it was found that the release of 1/2 and 1/1 was higher than 2/1. After comprehensive consideration, the final choice of mass ratio was 1/1 (Figure S11, Supplementary Materials). The brain of AD mice contains a large amount of copper ions, and D-Pen is an excellent copper ion chelating reagent. Therefore, we simulated the release of D-Pen under physiological conditions with and without copper ions. The results show that the release rate of D-pen was significantly increased under the induction of copper ions (Figure S12, Supplementary Materials).

In order to evaluate the in vitro release properties of DHQ and D-pen, the release of both drugs was studied in a Cu^2+^ + PBS environment at 37 °C. DD@MOF was dispersed in PBS for DHQ and D-pen release. It was placed in a constant temperature shaker at 37 °C and a portion of the liquid was removed at regular intervals to be used as a subsequent drug content assay. The drug release was monitored for a total of 72 h and the removed samples were tested for content by UV–vis and HPLC, respectively. The release curves of DHQ and D-pen show that 4 h ago, the release rate of D-pen was higher than that of DHQ, and D-pen regulates the microenvironment. Four hours later, the release rate of DHQ increased and the drug treatment began (Figure 1f). We further fitted the released data using zero-order, first-order, Higuchi, and Korsmeyer–Peppas (KP) kinetic models, respectively. The fitting results are summarized in Table S2. The results show that the first-order model provides the highest goodness of fit for both drugs (R^2^ = 0.993 and R^2^ = 0.983), indicating that the release rates of D-pen and DHQ are proportional to the remaining amount of drug in the system and that the process is primarily controlled by diffusion. The release curves are observed to exhibit a rapid initial increase followed by a plateau, which is characteristic of diffusion-controlled release. The Korsmeyer–Peppas model yields lower R^2^ values (0.726 and 0.857), and the release exponent n for both drugs is far below 0.5, further confirming that the release mechanism follows Fickian diffusion, consistent with the first-order model conclusion. In contrast, the zero-order and Higuchi models provide poorer fits, suggesting that the release is neither zero-order nor ideal matrix diffusion. Consequently, the drug release from DD@MOF is best described by first-order kinetics, with diffusion from the carrier being the dominant mechanism. The reason for the fast and slow difference in the release of D-pen and DHQ is that (1) DHQ has a stronger Π-Π interaction with MOF; (2) D-pen is a hydrophilic substance, which is more likely to form hydrogen bonds with water and has a stronger affinity for water; and (3) the presence of copper ions induced the accelerated release of D-pen.

### 2.3. Antioxidant Properties of DD@MOF

The antioxidant activity of DD@MOF was systematically evaluated through electron paramagnetic resonance (EPR) spectroscopy coupled with standard radical scavenging assays. Two well-established methodologies were employed: (i) the DPPH (1,1-diphenyl-2-picrylhydrazyl) radical assay, where the nitrogen-centered free radical exhibits characteristic absorption at 516 nm that diminishes upon interaction with antioxidants; and (ii) the ABTS [2,2′-azino-bis(3-ethylbenzothiazoline-6-sulfonic acid)] decolorization assay, in which the cationic radical (ABTS+) demonstrates a distinct absorption maximum at 753 nm that attenuates in the presence of radical scavengers [29,30,31]. Comparative analysis revealed that DHQ, DHQ@MOF, and DD@MOF exhibit significant antioxidant capacity, demonstrating superior free radical scavenging efficacy relative to the conventional antioxidant ascorbic acid (ASA), concurrently (Figures S13–S15, Supplementary Materials). We simulated the conditions containing copper ions to explore whether the antioxidant capacity of DHQ would be affected by metal ions. The results show that the antioxidant capacity of DHQ was inhibited, but the antioxidant capacity of DHQ was restored in the presence of D-pen (Figure 1g–i). To carefully evaluate the antioxidant properties of DHQ@MOF and DD@MOF, we further fitted and calculated their IC_50_ values, with the results shown in Table S3. Analysis revealed that the dual-drug-loaded DD@MOF exhibited the strongest antioxidant activity in both ABTS and DPPH assays, with IC_50_ values of 8.711 and 6.953 μg/mL, respectively, confirming that dual-drug synergy significantly enhanced antioxidant capacity. The antioxidant performance was further analyzed under simulated pathological conditions with the addition of copper ions. The IC_50_ values for Cu^2+^ + DD@MOF were 13.320 and 7.415 μg/mL, while those for Cu^2+^ + DHQ@MOF were 42.733 and 33.510 μg/mL. Although the IC_50_ of Cu^2+^ + DD@MOF slightly increased compared to the copper-free condition, it remained far superior to that of Cu^2+^ + DHQ@MOF, indicating that DD@MOF can maintain high activity even in a copper-overloaded environment. This also further demonstrates that D-pen first chelates excess Cu^2+^, enabling DD@MOF to exhibit superior antioxidant performance.

### 2.4. DD@MOF Ameliorate Neuroinflammation Symptoms In Vivo

Before conducting in vivo treatment in mice, to verify whether DD@MOF can penetrate the blood–brain barrier (BBB), we administered the drug via intraperitoneal injection and quantitatively measured the Fe^3+^ levels in mouse brain tissue at different time points using inductively coupled plasma mass spectrometry (ICP-MS). As shown in Figure S16, the brain Fe^3+^ concentration increased in a time-dependent manner, rising from 0.39 μg/mL at 2 h to 0.54 μg/mL at 24 h. Compared to the baseline level of 0.36 μg/mL at 0 h, this represents a net increase of approximately 0.18 μg/mL. This upward trend indicates that DD@MOF can enter and accumulate in brain tissue after administration. These results indirectly support the ability of DD@MOF to cross the BBB, providing a basis for its potential use in subsequent brain therapy.

**Figure 2:**
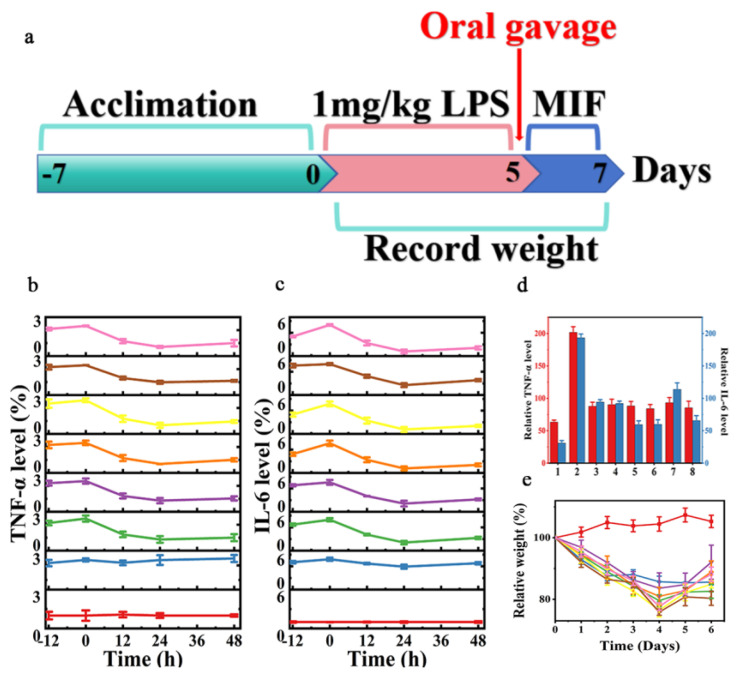
DD@MOF ameliorate neuroinflammation symptoms in vivo. (**a**) Scheme of LPS-induced neuroinflammation and treatment with DD@MOF (MIF: monitoring of inflammatory factors). (**b**) Relative TNF-α levels. The data are presented as mean ± SD (*n* = 8) (red: control; blue: LPS; green: LPS + DHQ; purple: LPS + DD; orange: LPS + DHQ@MOF; yellow: LPS + DD@MOF; brown: LPS + Cu^2+^ + DHQ@MOF; pink: LPS + Cu^2+^ + DD@MOF). (**c**) Relative IL-6 levels. The data are presented as mean ± SD (*n* = 8) (red: control; blue: LPS; green: LPS + DHQ; purple: LPS + DD; orange: LPS + DHQ@MOF; yellow: LPS + DD@MOF; brown: LPS + Cu^2+^ + DHQ@MOF; pink: LPS + Cu^2+^ + DD@MOF). (**d**) Comparative images of inflammatory factor levels in the groups at 48 h. The data are presented as mean ± SD (*n* = 8) (1: Control; 2: LPS; 3: LPS + DHQ; 4: LPS + DD; 5: LPS + DHQ@MOF; 6: LPS + DD@MOF; 7: Cu^2+^ + DHQ; 8: Cu^2+^ + DD@MOF). (**e**). Images of weight changes in the LPS-induced neuroinflammation experiment. The data are presented as mean ± SD (*n* = 8) (red: PBS; blue: LPS; green: LPS + DHQ; purple: LPS + MOF; orange: LPS + DHQ@MOF; yellow: LPS + DD@MOF; brown: LPS + Cu^2+^ + DHQ@MOF; pink: LPS + Cu^2+^ + DD@MOF).

Based on the encouraging antioxidant properties of DD@MOF, we evaluated the in vivo therapeutic efficacy of DD@MOF in an LPS-induced neuroinflammatory mouse model. A mouse model of neuroinflammation was constructed by lipopolysaccharide (LPS) intraperitoneal injection (IP) of ICR mice. The DHQ and D-PEN were prepared as a 40 mg/kg solution of normal saline, while the DD@MOF was prepared as an 80 mg/kg solution of normal saline. After ultrasonic treatment, 200 μL was injected intraperitoneally into the mice. The weight of the mice was approximately 20 g. Inflammatory factors TNF-α and IL-6 were closely associated with neuroinflammation (Figure 2a). In mice without copper ions, DHQ, DD, DHQ@MOF, and DD@MOF were all effective in reducing TNF-α and IL-6 in the mouse brain during the pre-treatment period (Figure 2b,c; Figure S17, Supplementary Materials). Surprisingly, in the LPS mouse model containing copper ions in vivo, the effect of DHQ was significantly inhibited compared with that without copper ions, but DHQ could play a better role in the presence of D-pen (Figure 2d). This indicates that D-pen can prevent DHQ from binding to copper ions so that DHQ can play a better role in controlling the level of inflammatory factors in the body to improve neuroinflammation. In addition, LPS-induced neuroinflammation caused weight loss in mice. After DD@MOF treatment, the mice returned to a stable body weight (Figure 2e). In general, when there was no copper ion in mice, the effects of DHQ and DD@MOF were not much different. However, in the presence of copper ions, the effect of DHQ was significantly inhibited compared with the DD@MOF group.

### 2.5. DD@MOF Biosafety

We evaluated the biosafety of DD@MOF. First, 1 mg of AAPD, Fe^3+^, and MOF were dissolved in 1 mL of physiological saline. The solution was shaken and then injected intraperitoneally into mice at a dose of 200 μL for consecutive 3 days. Potential in vivo toxicity was assessed by detecting changes in inflammatory factor levels in mice for 3 days and changes in body weight for 7 days. The results show low levels of inflammatory factors in all mice (Figures S18 and S19, Supplementary Materials). There was no significant difference in body weights (Figure S20, Supplementary Materials), which indicated that the toxicity of all materials was negligible. Subsequently, the results of in vitro hemolysis tests demonstrated that DD@MOF had superior hemocompatibility and did not cause mouse erythrocytes rupture (Figure S21, Supplementary Materials). In addition, we evaluated the biosafety of all materials in the heart, liver, spleen, lungs, and kidneys. The images showed no significant toxicity of all materials to major organs (Figure S22, Supplementary Materials).

### 2.6. DD@MOF Ameliorate AD Symptoms In Vitro

**Figure 3:**
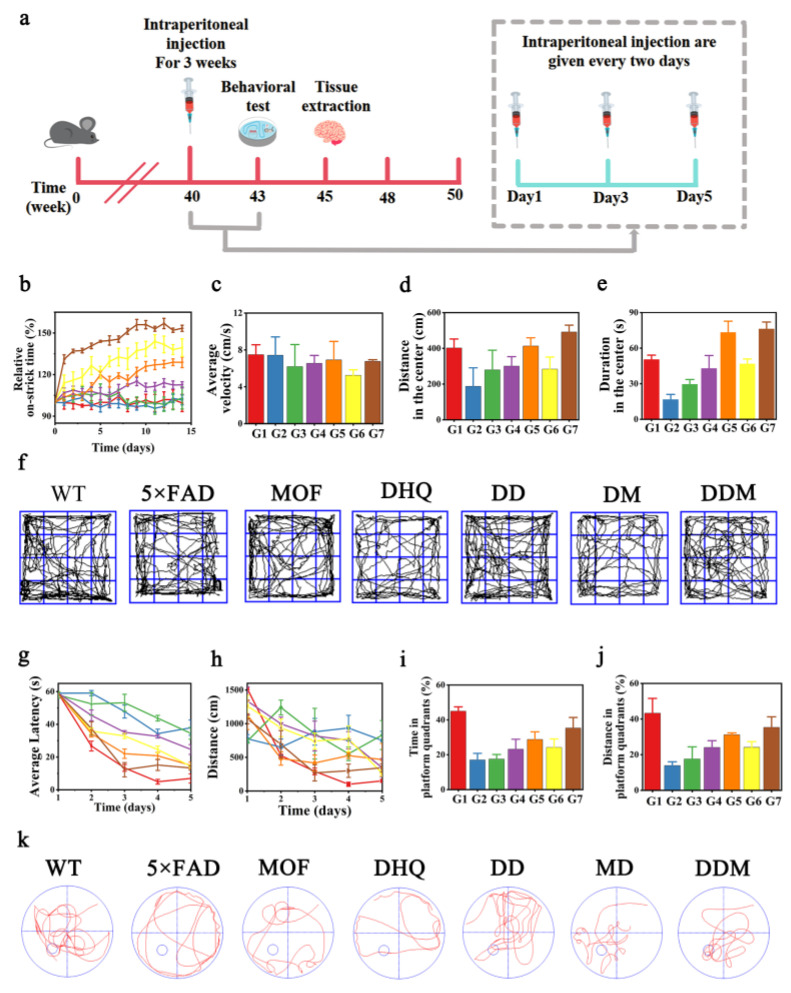
Behavioral tests of 5 × FAD mice treated. (**a**) Timeline of the animal experiment. (**b**) Time of the mice dropping the rota-rod. The data are presented as mean ± SD (*n* = 8). (red: WT; blue: 5 × FAD; green: 5 × FAD + MOF; purple: 5 × FAD + DHQ; orange: 5 × FAD + DD; yellow: 5 × FAD + DHQ@MOF; brown: 5 × FAD + DD@MOF). (**c**) average latency of 5 × FAD mice in the open field test. The data are presented as mean ± SD (*n* = 8). (**d**) distance in the center area by 5 × FAD mice in the OFT. The data are presented as mean ± SD (*n* = 8). (**e**) duration in the center by 5 × FAD mice in the OFT. The data are presented as mean ± SD (*n* = 8). (**f**) Representative tracks of mice from seven groups in the OFT. (**g**) Curve of average latency during the acquisition phase in MWM. The data are presented as mean ± SD (*n* = 8). (red: WT; blue: 5 × FAD; green: 5 × FAD + MOF; purple: 5 × FAD + DHQ; orange: 5 × FAD + DD; yellow: 5 × FAD + DHQ@MOF; brown: 5 × FAD + DD@MOF). (**h**) Curve of distance during the acquisition phase in MWM. The data are presented as mean ± SD (*n* = 8). (red: WT; blue: 5 × FAD; green: 5 × FAD + MOF; purple: 5 × FAD + DHQ; orange: 5 × FAD + DD; yellow: 5 × FAD + DHQ@MOF; brown: 5 × FAD + DD@MOF). (**i**) Time in the platform quadrant (%) on day 6 in MWM. The data are presented as mean ± SD (*n* = 8). (**j**) distance in the platform quadrant (%) on day 6 in MWM. The data are presented as mean ± SD (*n* = 8). (**k**) Representative tracks of mice from seven groups in MWM. (G1: WT; G2: 5 × FAD; G3: 5 × FAD + MOF; G4: 5 × FAD + DHQ; G5: 5 × FAD + DD; G6: 5 × FAD + DHQ@MOF; G7: 5 × FAD + DD@MOF unified in the image).

The therapeutic regimen for 5 × FAD mice initiated at 10 months of age, involving intraperitoneal injections administered once every two days over a three-week period. Subsequent to this treatment, comprehensive behavioral assessments were conducted to evaluate neurobehavioral functions, followed by tissue extraction for downstream experimental analyses (Figure 3a). We established seven groups as follows: (1) WT mice; (2) 5 × FAD mice (3) 5 × FAD mice treated with MOF; (4) 5 × FAD mice treated with DHQ; (5) 5 × FAD mice treated with DHQ and D-pen; (6) 5 × FAD mice treated with DHQ@MOF; and (7) 5 × FAD mice treated with DD@MOF. Throughout the treatment period, we measured the body weight changes of the mice every day and observed that the body weight changes of the mice in each group were not significant, indicating that the administration of MOF and DD@MOF did not have a toxic effect on the mice (Figure S23, Supplementary Materials). As shown in Figure S24, during the administration period, we measured the levels of white blood cells, lymphocytes, red blood cells, and neutrophils in the mouse blood, as well as liver and kidney function indicators (alanine aminotransferase, aspartate aminotransferase, urea, creatinine). The results show that all parameters remained within the normal physiological range. These data indicate that at the experimental dose and duration, DD@MOF did not cause significant liver, kidney, or systemic toxicity, demonstrating good safety. To comprehensively evaluate cognitive and motor functions, we conducted a series of behavioral tests. The rotarod test showed that DD@MOF treatment significantly improved the motor coordination of AD model mice (Figure 3b). In the open field test, there was no significant difference in the average speed among the groups (Figure 3c), but DD@MOF treatment significantly increased the distance traveled and time spent in the central area (Figure 3d,e), suggesting an alleviation of anxiety-like phenotypes. In the Morris water maze test, there was no significant difference in swimming speed among the groups during the training period. Repeated-measures analysis of the 5-day training data revealed that DD@MOF treatment significantly shortened the latency and distance to reach the platform and improved the learning rate (Figure 3g,h). In the final probe trial, compared with the 5 × FAD group, the DD@MOF-treated group showed significantly increased time spent, distance traveled in the target quadrant, and number of platform crossings (Figure 3i–k), indicating improved spatial memory retention.

### 2.7. DD@MOF Effect of Nanoparticles In Vivo

**Figure 4:**
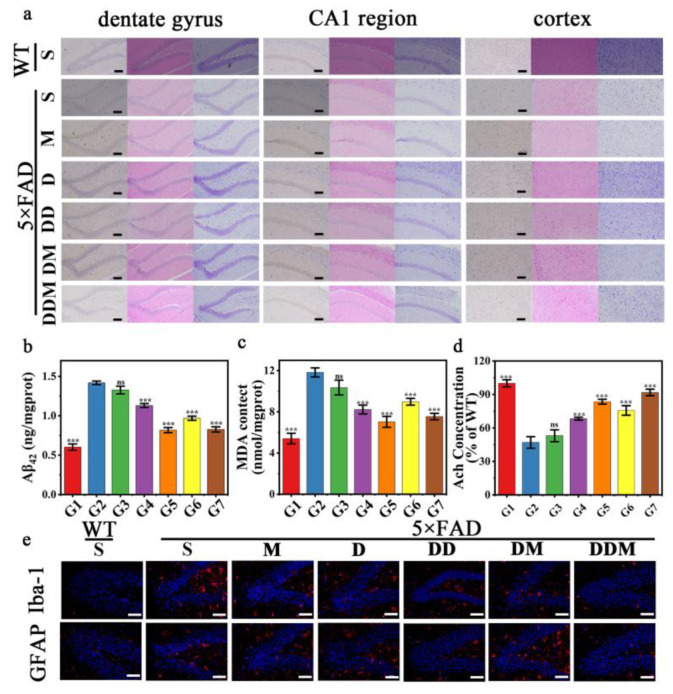
DD@MOF effect of nanoparticles in vivo. (**a**) Immunohistochemical staining (brown), HE (pink), and Nissl staining (purple) in the cortex and hippocampus of mice after treatment with different preparations. Bar = 200 μm. (**b**) Quantitative analysis of Aβ1–42 in the hippocampus of mice after treatment with different preparations, determined by an ELISA assay. The data are presented as mean ± SD (*n* = 8). *** *p* = 0.0001. ns = no significant difference. (**c**) Quantitative MDA content in the hippocampus of mice after treatment with different preparations. The data are presented as mean ± SD (*n* = 8). *** *p* = 0.0001. ns = no significant difference. (**d**) Quantitative Ach Concentration in the hippocampus of mice after treatment with different preparations. The data are presented as mean ± SD (*n* = 8). *** *p* =0.0001. ns = no significant difference. (**e**) Immunohistochemical fluorescence staining of Iba-1 and GFAP in the brain of mice treated with different preparations. Bar = 50 μm (S: saline; M: MOF; D: DHQ; DD: DHQ + D-pen; DM: DHQ@MOF; DDM: DD@MOF; G1: WT; G2: 5 × FAD; G3: 5 × FAD + MOF; G4: 5 × FAD + DHQ; G5: 5 × FAD + DD; G6: 5 × FAD + DHQ@MOF; G7: 5 × FAD + DD@MOF unified in the image). (the blue color represents Iba-1 and GFAP, while the red color represents the cell nucleus).

The extracellular accumulation of amyloid-beta peptides (Aβ) constitutes a defining neuropathological feature of Alzheimer’s disease, manifesting as senile plaque formation in cortical regions. Thus, reducing the Aβ burden is considered to be one of the potential strategies in AD therapy. To assess the Aβ situation in the brains of mice from each group after treatment, all mice were euthanized after 3 weeks of treatment and their brains were collected for immunohistochemical staining. The stained images showed that DD@MOF, DHQ@MOF, DD, and DHQ treatments all reduced Aβ levels in the brains of 5 × FAD mice (Figure 4a). Quantitative analysis of Aβ levels in murine brain tissue was performed using enzyme-linked immunosorbent assay (ELISA), enabling precise measurement of amyloid-beta peptide concentrations (Figure 4b). According to the results, we can draw a conclusion that the DD@MOF, DHQ@MOF, DD, and DHQ have the ability to clear the Aβ plaques in the AD mice. Encouragingly, DD @ MOF has the best scavenging effect on Aβ.

Oxidative stress plays a significant pathogenic role in the onset and progression of neurodegenerative disorders, particularly Alzheimer’s disease (AD). Malondialdehyde (MDA) levels serve as a reliable biomarker for assessing the extent of reactive oxygen species (ROS)-mediated tissue damage. Therefore, the MDA content in the brain was detected using assay kits (Figure 4c). The MDA content in the brain of AD mice was significantly higher than those of WT mice; however, these symptoms were significantly relieved in mice receiving DD@MOF. Acetylcholine plays a key role in the regulation of learning and memory. Impaired cholinergic transmission can lead to cognitive decline associated with aging and dementia. We used the kit to detect the content of Ach in brain tissue. The results show that the content of Ach in the brain of AD mice was significantly lower than that of WT mice. However, it is gratifying that the content of Ach in the brain of AD mice after DD@MOF treatment is close to that of WT mice (Figure 4d). The standard curve was fitted using an exponential model (y = 4.310 − 4.244 × e^−0.003x^) to describe the relationship between different concentrations of DD@MOF and acetylcholinesterase (AChE) activity, with excellent fitting performance (R^2^ = 0.999) (Figure S25). The curve shows that absorbance increases with DD@MOF concentration, rising rapidly in the low-concentration range (0–50 nmol/L) and gradually leveling off in the high-concentration range (>100 nmol/L), indicating that the enzymatic reaction approaches saturation. This curve can be used for the precise determination of AChE activity in unknown samples. Beyond the well-documented pathological hallmarks of amyloid-beta (Aβ) accumulation and cholinergic system dysfunction, emerging evidence highlights neuroinflammatory processes as a critical third pillar in Alzheimer’s disease (AD) pathogenesis. Histopathological analyses of AD model mice reveal extensive glial activation, demonstrated by intense immunohistochemical labeling of Iba-1-positive microglia and GFAP-expressing astrocytes throughout affected brain regions (Figure 4e). However, fewer activated microglia and astrocytes were observed in AD mice after the treatment with DD@MOF. Moreover, the expression of TNF-α and IL-6 in the brain of AD mice was detected using ELISA kits (Figure S27, Supplementary Materials). The levels of TNF-α and IL-6 were 2.93 and 2.48 higher, respectively, in the AD mice than those in the WT group [32,33,34,35]. The TNF-α and IL-6 contents decreased significantly after the DD@MOF and DHQ@MOF treatment, attributed to the anti-inflammatory effects mediated by DHQ (Figures S27 and S28, Supplementary Materials). Meanwhile, the anti-inflammatory effect of DD@MOF was better than that of DHQ@MOF because D-pen pre-chelated copper ions, avoiding the combination of DHQ and copper ions, making DHQ play a better role. In addition, we assessed the biosafety in the heart, liver, spleen (Figure S29, Supplementary Materials). To verify the copper ion chelation mechanism, we measured the copper ion content in mouse brains under different treatment conditions using ICP, with the results shown in Figure S30. In the brains of WT mice, the copper ion content remained at a low level. In contrast, copper ions accumulated significantly in the brains of AD model mice, reaching 1.4 times the level of WT mice. After DHQ@MOF treatment, the brain copper ion level decreased only slightly by 5.80%. In contrast, after DD@MOF treatment, the copper ion level was significantly reduced, with a decrease of 21.58%. This result clearly demonstrates that DD@MOF exhibits a more pronounced effect in chelating and clearing excess copper ions from the brain.

## 3. Discussion

Recent studies have highlighted polyphenols as promising therapeutic candidates for Alzheimer’s disease (AD), primarily through their dual capacity to suppress amyloid-beta (Aβ) fibrillization and scavenge reactive oxygen species (ROS). Nevertheless, the therapeutic potential of these compounds is significantly compromised by the elevated concentrations of metal ions within amyloid plaques. To address this limitation, we developed DD@MOF, a metal–organic framework-based delivery system that enables sequential drug release through precisely engineered carrier–drug molecular interactions.

In the sequential release system, the metal chelator D-pen, which is released first, theoretically has the potential to chelate Cu^2+^ in amyloid plaques. Subsequently, over time, DHQ was released to play a therapeutic role. This phased release strategy significantly enhances the therapeutic efficacy of polyphenolic agents against Alzheimer’s disease. In addition, this drug release system not only can Cu^2+^ and eliminate excess ROS, it also has excellent antioxidant properties and good biocompatibility. This study presents an innovative approach to address metal ion interference in polyphenol-based therapies, with potential applications extendable to other drug delivery systems affected by metal ion interactions.

## 4. Materials and Methods

### 4.1. Reagents and Materials

Hexahydrate of ferric chloride (FeCl_3_·6H_2_O, purity ≥ 99%) was purchased from Shanghai Aladdin Biochemical Science and Technology Co. (Shanghai, China). 2-Aminoterephthalic acid (C_8_H_7_NO_4_, AAPD, purity 98%) was purchased from Shanghai Macklin Biochemical Technology Co. (Shanghai, China). *N*,*N*-Dimethylformamide (DMF, purity ≥ 99.5%) was purchased from Tianjin Jindong Tianzheng Fine Chemical Reagent Factory (Tianjin, China). Anhydrous ethanol (CH_3_CH_2_OH, purity ≥ 99.7%) and anhydrous methanol (CH_3_OH, purity ≥ 99.5%) were purchased from Tianjin Xinbute Chemical Co. (Tianjin, China); D-penicillaminel (D-pen, purity 99%), potassium persulfate (K_2_S_2_O_8_, purity 99.9%) and 2,2-Diphenyl-1-picrylhydrazyl (DPPH, purity ≥ 98.5%) were purchased from Shanghai McLean Biochemistry Technology Co. (Shanghai, China). Dihydroquercetin (DHQ, purity ≥ 90%) was purchased from Aladdin Scientific Corp. (Shanghai, China). Ascorbic acid (ASA, 99% purity) was purchased from Beijing Bo Aosun Biotechnology Co. (Beijing, China). 2,2′-Azino-bis(3-ethylbenzothiazoline-6-sulfonic acid) diammonium salt (ABTS, 99.67% purity) was purchased from Shandong Skeje Biotechnology Co. (Jinan, China).

### 4.2. Characterization

KBr particles were analyzed by Fourier Transform Infrared Spectroscopy (FTIR) in the wavelength range of 4000–400 cm^−1^ using a Nicolet IS50 infrared spectrometer (Thermo Fisher Scientific, Waltham, MA, USA). Scanning electron microscopy (SEM) tests were carried out with a JEOL-JSM-7600 scanning electron microscope instrument (Akishima, Japan) with an accelerating voltage of 5 kV. Powder X-ray diffraction (PXRD) tests were carried out with a Dmax2200PC diffractometer (Nippon Rikyo Corporation, Japan) with a scanning range of 5–80° (2θ) using Cu-Ka radiation, 40 kV, 200 mA, and a scanning rate of 5° min^−1^. Adsorption–desorption isotherms of nitrogen were measured on a Quantachrome Autosorb-iQ2 gas adsorption instrument (Anton Paar, Graz, Austria) at 77 K with a relative pressure of 0–1 bar. Thermogravimetric analysis (TGA) was carried out on a METTLER-TOLEDO TGA/DSC Model 3+ thermogravimetric analyzer (Zurich, Switzerland) in the temperature range of 30–800 °C with a heating rate of 10 °C/min under air conditions. High-performance liquid chromatography (HPLC) was tested on an Agilent Model 1200 chromatograph (Santa Clara, CA, USA) at 30 °C. EPR Bruker was tested on EMX plus-6/1 (Billerica, MA, USA).

### 4.3. Preparation of NH_2_-MIL-101(Fe)

In the microwave-assisted method, firstly, iron solution was prepared by dissolving FeCl_3_·6H_2_O (10 mmol) and acetic acid (1 mL) in 80 mL of dimethylformamide (DMF) under sonication, followed by the addition of 2-Aminoterephthalic acid (5 mmol) to the solution. The solution was then transferred into a 250 mL 4-neck round bottom flask and placed in microwave reactor and heated at 110 °C. After 45 min, the reactor was cooled down to room temperature and the precipitates were collected by centrifugation. The as-obtained products were further purified using DMF and methanol, respectively, and finally dried under vacuum at 60 °C overnight.

### 4.4. Preparation of DD@MOF

DHQ, D-pen, and NH_2_-MIL-101(Fe) in a mass ratio of 1:1:1 were mixed in anhydrous ethanol and stirred for 24 h. The precipitate and supernatant were then collected by centrifugation at 8000× *g*. It was washed with anhydrous ethanol, dried under vacuum at 80 °C, and used directly in the next step of drug release experiments. The encapsulation rate and drug loading of the two drugs were calculated by determining the DHQ and D-pen content in the collected supernatant. The drug content of the supernatant was determined using high-performance liquid chromatography (HPLC) to obtain the encapsulation rate and drug loading rate of DD@MOF. The encapsulation rates of DHQ and D-pen were 37.4% and 52.7%, and the drug loading rates were 25.8% and 29.6%, respectively.

### 4.5. Release of DD@MOF

In order to study the release of both DHQ and D-pen, the release of both drugs is studied in PBS at 37 °C. DD@MOF is dispersed in PBS for DHQ and D-pen release. It is placed in a constant temperature shaker at 37 °C, and a portion of the liquid is removed at regular intervals to be used as a subsequent drug content assay. The drug release is monitored for a total of 72 h, and the removed samples are analyzed for content by UV–vis and HPLC, respectively. The drug loading and in vitro release were quantitatively analyzed using a high-performance liquid chromatography (HPLC) system (Agilent 1260 Infinity II, or other model, Agilent Technologies, CA, USA). The chromatographic conditions were as follows: a Tilank C18 column (Guangzhou Filuomen Scientific Instrument Co., Ltd., Guangzhou, China) (50 mm × 4.6 mm, 5 μm); mobile phase A: aqueous solution containing 0.05% (*v*/*v*) formic acid; mobile phase B: acetonitrile solution containing 0.04% (*v*/*v*) formic acid; a linear gradient elution with a total run time of 10 min (0–5 min: phase A decreased linearly from 90% to 10%, phase B increased linearly from 10% to 90%; 5–10 min: phase A maintained at 10%, phase B at 90%); flow rate: 1.0 mL/min; column temperature: 30 °C; detection wavelength: 280 nm (using a diode array detector, DAD (Dalian Elite Analytical Instrument Co., Ltd., Dalian, China)); injection volume: 1.0 μL. The test samples were diluted with methanol to 0.1 mg/mL prior to injection.

### 4.6. Determination of Antioxidant Capacity of DD@MOF

To determine DPPH radical scavenging activity, DPPH solution was prepared with ethanol. We added 1000 μL of DPPH solution to 100 μL of MOF, DHQ, ASA, DHQ@MOF, DD@MOF, Cu^2+^ + DHQ@MOF, and Cu^2+^ + DD@MOF at different concentrations. We incubated it for 30 min at room temperature with shaking and mixing, and then we replaced the control with ethanol. The absorbance at 517 nm was measured. The DPPH radical scavenging activity was calculated as follows:

(1) $$DPPH\;scavenging\;activity\;\left(\%\right)\;=\;\left(Acontrol-Asample\right)\;÷\;\left(Acontrol\right)\;\times 100\;$$

To determine ABTS free radical scavenging activity, ABTS reagent at a concentration of 7 × 10^−3^ mol/L and potassium persulfate at 2.45 × 10^−3^ mol/L were mixed in a 1:1 volume, respectively. This was incubated for 12–16 h at room temperature, avoiding light, to make ABTS+· solution. Before use, the ABTS+· solution was diluted so that the absorbance value at 753 nm was 0.70 ± 0.02. Then, 2000 μL of diluted ABTS+· solution was added to 1000 μL of MOF, DHQ, ASA, DHQ@MOF, DD@MOF, Cu^2+^ + DHQ@MOF, and Cu^2+^ + DD@MOF at different concentrations. The reaction system was incubated at room temperature for 10 min. The absorbance of the reaction system at 753 nm was measured, and the control group was replaced by ethanol. The formula for calculating the radical scavenging activity of ABTS was as follows:

(2) $$ABTS\;scavenging\;activity\;\left(\%\right)\;=\;\left(Acontrol-Asample\right)÷\left(Acontrol\right)\times 100\;$$

where Acontrol and Asample are the absorbance values of the control and experimental groups, respectively.

### 4.7. In Vitro Hemolysis Test

To evaluate the biosafety of DD@MOF, mouse erythrocytes were obtained from AD mice. Mouse erythrocytes were collected from serum by centrifugation (3000 r min^−1^, 5 min) and then washed four times with PBS. The rinsed mouse erythrocytes were diluted with PBS to 1/5. Then, saline, H_2_O, and saline containing 50, 250, 500, 750, and 1000 μg mL^−1^ DD@MOF were co-incubated with 30 μL of mouse erythrocytes at 37 °C for 3 h, and whether the mouse erythrocytes were ruptured was observed.

### 4.8. Animal Model

The 5 × FAD mice (APP/PS1, C57BL/6) were purchased from Gempharmatech Co., Ltd. (Nanjing, China) (male, 10 months). Wild-type mice (male, 10 months) and ICR mice were purchased from Changchun Yise Laboratory Animal Technology Limited Liability Company (Changchun, China). The animals were housed in groups, and all mice were maintained in a 12 h light/dark cycle with temperature (24 ± 2 °C) and humidity (60 ± 5%) and provided with food and water randomly. All animal studies were conducted in accordance with the principles and procedures outlined in “Regulations for the Administration of Affairs Concerning Laboratory Animals”, approved by the National Council of China on 31 October 1988, and “The National Regulation of China for Care and Use of Laboratory Animals,” promulgated by the National Science and Technology Commission of China on 14 November 1988. All animal studies were supervised by the Committee of Northeast Normal University Institutional Animal Care and Use. All operations related to mice strictly comply with the relevant requirements of the “Experimental Animal Environment and Facilities” (GB14925-2010) [36] and the “Guidelines for Ethical Review of Experimental Animal Welfare” (GB/T 35892-2018) [37].

### 4.9. DD@MOF In Vivo Long-Acting Treatment Trial

Purchased male ICR mice were uniformly acclimated in a standard barrier environment for one week. Subsequently, a systemic inflammation model was established by intraperitoneal injection of lipopolysaccharide (LPS, at a dose of 1 mg/kg body weight) daily for five consecutive days. After successful modeling, the mice were randomly divided into 9 groups, with 8 mice per group, as follows: control group (injected with saline only), LPS model group, LPS + DHQ group, LPS + DD group, LPS + MOF group, LPS + DHQ@MOF group, LPS + DD@MOF group, LPS + Cu^2+^ + DHQ@MOF group, and LPS + Cu^2+^ + DD@MOF group.

Justification for Sample Size: The sample size of 8 mice per group (*n* = 8) was further determined based on preliminary experimental data, the previous literature on inflammation models, and statistical power analysis to ensure the ability to detect statistically significant differences in inflammatory cytokine levels among the groups.

Randomization: After the LPS modeling phase, a researcher not involved in subsequent drug administration and sample analysis randomly allocated the animals to the aforementioned 9 experimental groups based on the mice’s body weight and general condition to ensure baseline balance across groups and reduce selection bias.

Blinding: Blinding was implemented at key stages to reduce subjective bias. Specifically, the operators responsible for subsequent drug administration (interventions) and euthanasia/sample collection were aware of the group assignments. However, the personnel involved in sample processing, inflammatory cytokine level assays, and final data statistical analysis were blinded to the specific group identities of the samples until all analyses were completed.

Ethics Statement: All animal experiments were supervised by the Animal Health and Utilization Committee of Northeast Normal University. Authorization Number: 202502059. Approval date: 1 January 2025. All experimental procedures strictly adhered to international and national/regional guidelines for animal welfare and ethics and followed the “3R” principles (Replacement, Reduction, Refinement) to minimize the number of animals used and alleviate their suffering.

Experimental Intervention and Sample Collection: Following group assignment, for the respective intervention groups, DHQ, DD, MOF, DHQ@MOF, and DD@MOF were each dissolved in saline at a dose of 40 mg/kg, ultrasonicated, and administered via a single intraperitoneal injection. The control and LPS model groups received an equivalent volume of saline. At specific time points post-administration, a corresponding number of mice were randomly euthanized from each group. Brain tissue samples were collected for subsequent analysis of inflammatory cytokines (e.g., TNF-α, IL-6).

### 4.10. DD@MOF Therapeutic Experiments on 5 × FAD Mice

All mice were acclimated to the environment for 7 days under standard housing conditions before the start of the experiment. This study used APP/PS1 transgenic mice as an Alzheimer’s disease (AD) model, with wild-type (WT) mice of the same background serving as controls. Based on preliminary experimental data and previous literature, a sample size of *n* = 8 per group was set to detect significant differences in primary behavioral indicators (such as the Morris water maze) while controlling for individual variation. APP/PS1 mice were randomly divided into 6 groups: the AD model group, AD + MOF group, AD + DHQ group, AD + DD group, AD + DHQ@MOF group, and AD + DD@MOF group. Concurrently, WT mice were designated as the wild-type control group (WT group), resulting in a total of 7 groups with 8 mice each.

To investigate the effects of DD@MOF on the AD model, drug interventions were administered to the corresponding groups: DHQ, DD, MOF, DHQ@MOF, and DD@MOF were each dissolved in phosphate-buffered saline (PBS) and administered via intraperitoneal injection at a dose of 10 mg/kg (in a volume of 200 μL), once every two days for a total of 3 weeks. After the completion of drug administration, all mice underwent a series of behavioral tests, including the rotarod test (to assess motor coordination), the open field test (to assess spontaneous activity and anxiety-like behavior), and the Morris water maze test (to assess spatial learning and memory abilities).

### 4.11. In Vivo Toxicity Studies

All mice were acclimated to standard housing conditions for 7 days prior to the start of the experiment. This study utilized APP/PS1 transgenic mice as an Alzheimer’s disease (AD) model, with wild-type (WT) mice of the same genetic background serving as controls.

Experimental Groups and Procedure: APP/PS1 transgenic mice and wild-type mice were randomly divided into the following 7 groups, with 8 mice per group: AD model control group, AD + AAPD group, AD + Fe^3+^ group, AD + DD group, AD + NH_2_-MIL-101(Fe) group, AD + DD@MOF group, and WT control group. For three consecutive days, once daily, the respective groups received intraperitoneal injections of saline solutions containing AAPD, Fe^3+^, DD, NH_2_-MIL-101(Fe), or DD@MOF (or an equal volume of saline as a control). Body weight changes were monitored during the administration period. After the final administration, blood samples were collected, and changes in the levels of serum inflammatory cytokines (such as TNF-α and IL-6) were measured to preliminarily assess systemic inflammatory responses. Subsequently, the mice were euthanized, and their major organs (heart, liver, spleen, lungs, and kidneys) were harvested. These organs were fixed, embedded, sectioned, and stained with H&E. Histomorphological changes in each organ were observed under light microscopy to evaluate the biosafety of the drugs.

### 4.12. Immunohistochemical Staining

Mouse brains were fixed in 10% paraformaldehyde and embedded in paraffin, and paraffin blocks were cut into 4 μm sections, deparaffinized with xylene, and rehydrated with ethanol. After blocking for 1 h, brain tissues were incubated with anti-Aβ antibody at 4 °C overnight. Sections were treated with AEC substrate chromogen to visualize the immune complexes. Corresponding images were acquired and analyzed using a microscope with a magnification of ×20.

### 4.13. H&E Staining of Major Organs

The hearts, livers, spleens, lungs, and kidneys of mice were paraffin-embedded and cut into thick sections for histological analysis. Sections were stained with hematoxylin and eosin (H&E) and visualized with a light microscope.

### 4.14. Statistical Analysis

All experimental data were expressed as mean ± standard deviation (SD). Significance analysis between different groups was performed by one-way analysis of variance (ANOVA). Significant difference analysis of data was expressed as *** *p* = 0.0001, **** *p*< 0.0001.

## Data Availability

The raw data supporting the conclusions of this article will be made available by the authors on request.

## Supplementary Materials

The following supporting information can be downloaded at https://www.mdpi.com/article/10.3390/molecules31010111/s1. Transmission electron microscopy (TEM) and N_2_ adsorption-desorption isotherms of DD@MOF; Fourier-transform infrared (FTIR) spectroscopy, thermogravimetric analysis (TGA), X-ray diffraction (XRD), ultraviolet-visible (UV-Vis) spectroscopy, and electron paramagnetic resonance (EPR) spectra of relevant samples; biological safety assessment (e.g., cell viability, hemolysis assay); and hematoxylin and eosin (H&E) staining images of major organs (heart, liver, spleen, lung, kidney) (PDF).
