# Supplementary material for: D-Penicillamine/Dihydroquercetin Dual-Loaded Metal–Organic Framework as a Microenvironment Copper Regulator for Enhancing the Therapeutic Efficacy of Polyphenolic Antioxidant in Alzheimer’s Disease
Source: Molecules. 2025 Dec 28;31(1):111. doi: 10.3390/molecules31010111 (PMC12787126; doi:10.3390/molecules31010111)
Supplement: Supplementary file 1 [file molecules-31-00111-s001.zip › molecules-4010264-supplementary.pdf]

Supporting Information

D-penicillamine/Dihydroquercetin Dual-loaded Metal-Organic Framework as a Microenvironment Copper Regulator for Enhancing the Therapeutic Efficacy of Polyphenolic Antioxidant in Alzheimer's disease

Xuhan Wu,Gang Huang, Licong Chen, Yiling Xie, Qi Ding, Enpeng Xi, Yun Zhao, Nan Gao,*

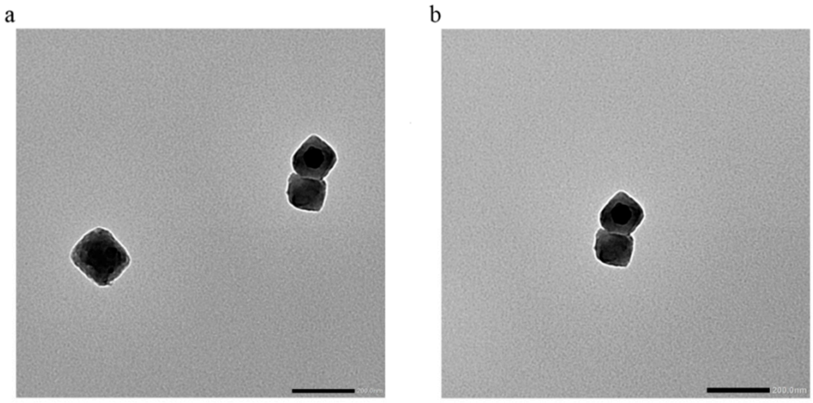

Figure S1. Morphology of materials. a) TEM for NH_2-MIL-101(Fe). b) TEM for DD@MOF.

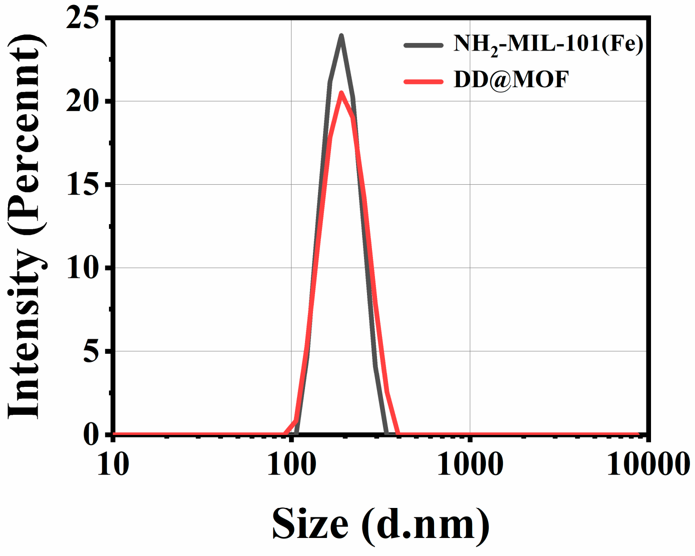

Figure S2. Hydrodynamic particle size of MOF and DD@MOF.

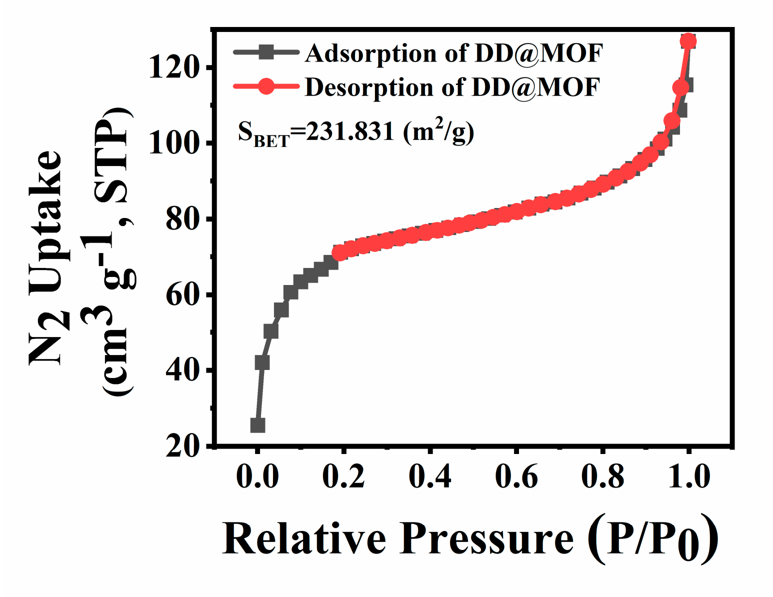

Figure S3. N_2 adsorption-desorption isotherms of DD@MOF

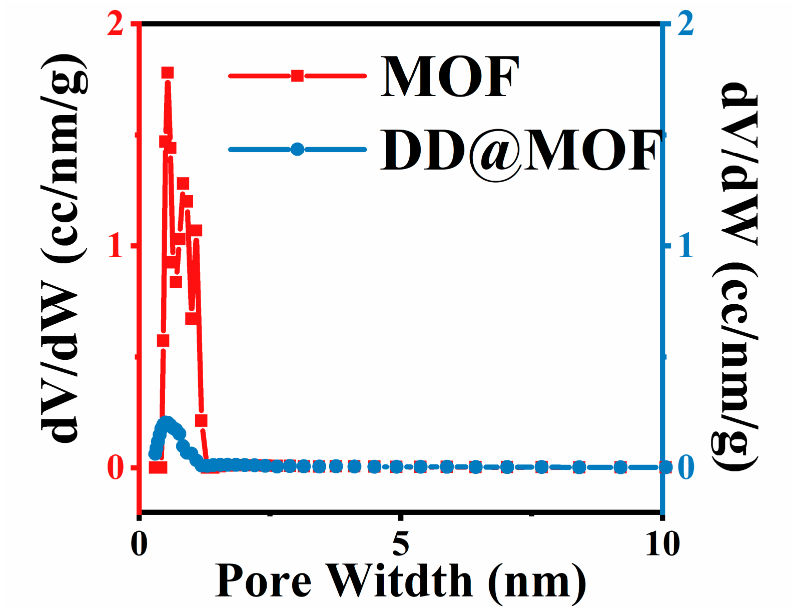

Figure S4. Pore size distribution of MOF and DD@MOF

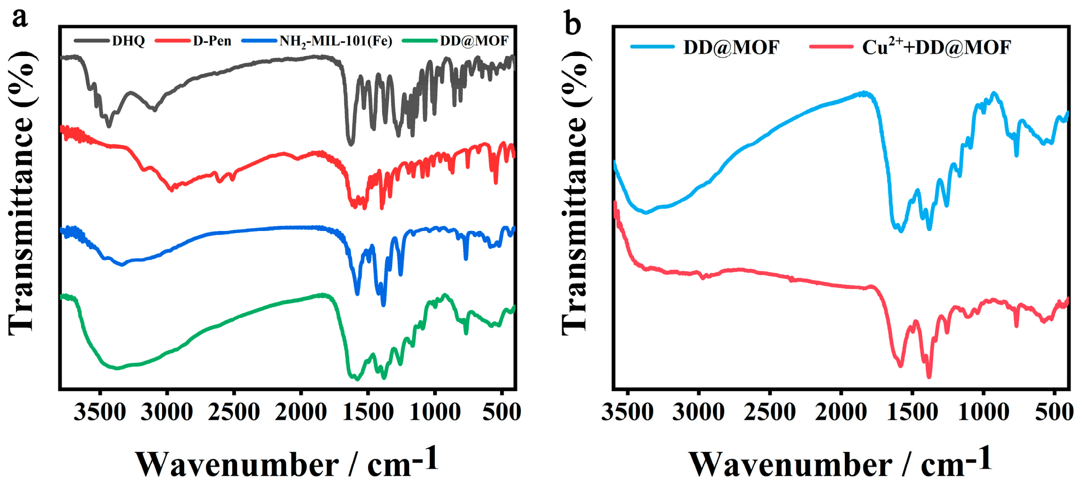

Figure S5. FTIR spectra of DHQ, D-pen, MOF, DD@MOF, Cu^{2+}+DD@MOF

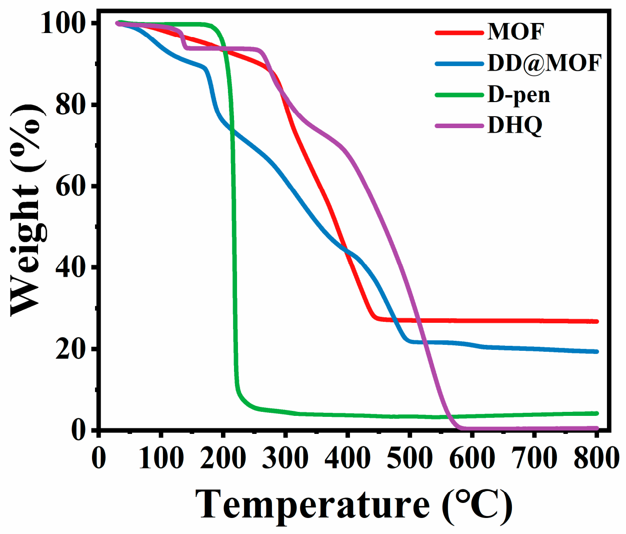

Figure S6. TGA of DHQ; D-pen; MOF and DD@MOF.

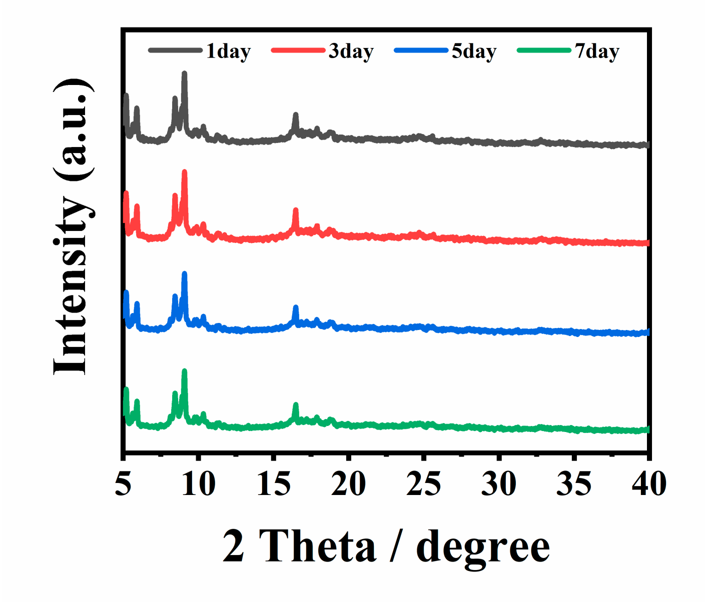

Figure S7. XRD: Stability of the DD@MOF in PBS for 7days.

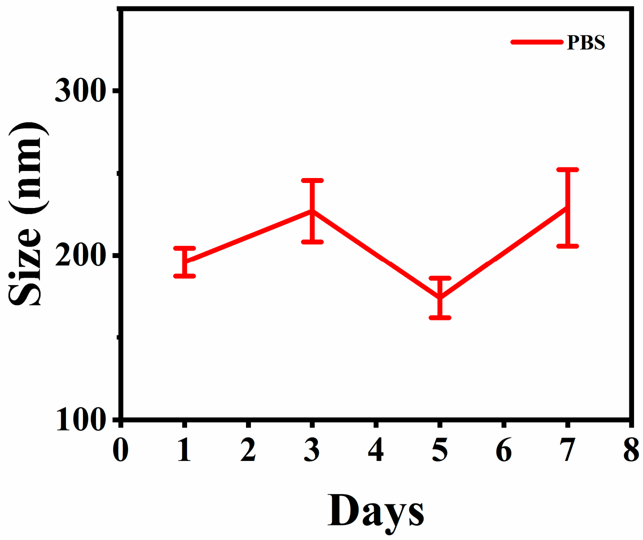

Figure S8. Hydrodynamic particle size of the materials in PBS for 7days.

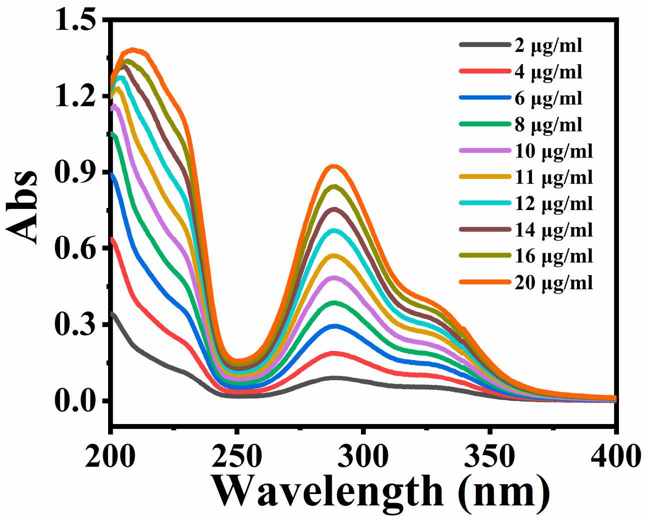

Figure S9. UV-Vis absorbance spectrum of DHQ in H_2O with different concen trations

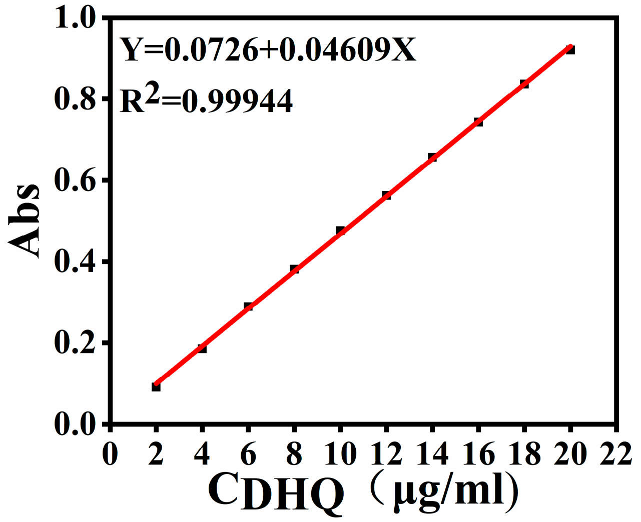

Figure S10. the standard curve of DHQ

Table S1. The DLCs and DLEs of different weight rations of MOF and DHQ.

Mass ration of NH_2-MIL-101(Fe) and DHQ	DLC (%)	DLE (%)
2:1	27.6%	39.6%
1:1	25.8%	49.7%
1:2	29.9%	43.6%

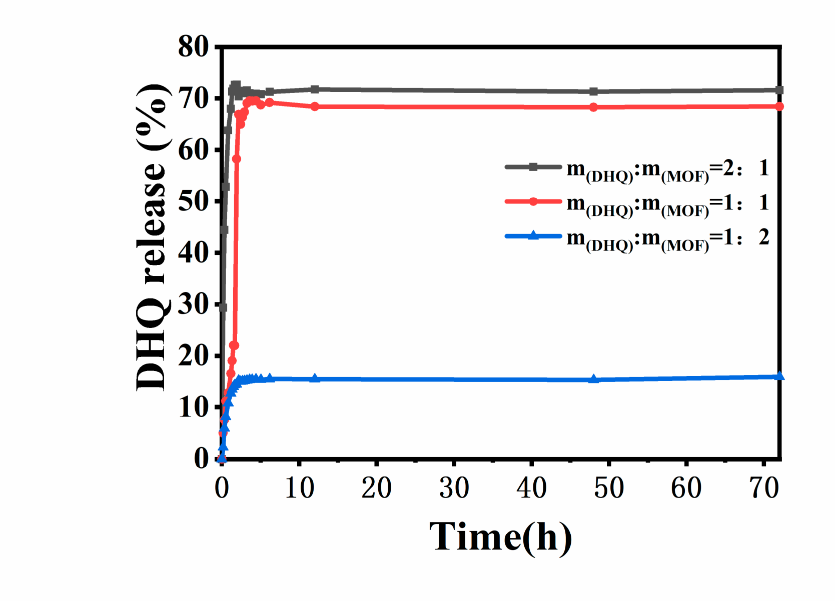

Figure S11. Release rates of DHQ@MOF at different weight ratios during the 24 hours.

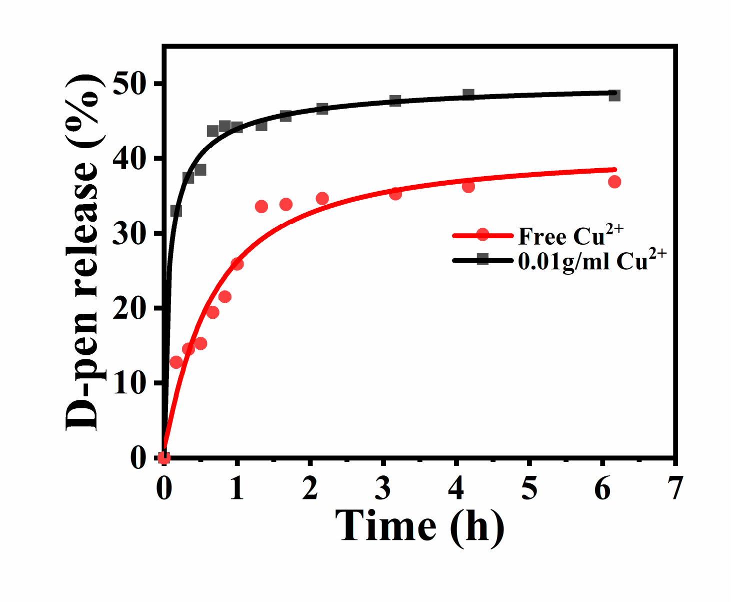

Figure S12. Release rates of D-pen @MOF in the environment with and without copper ions during the 7 hours.

Table S2. The drug release kinetics model results of DD@MOFD in PBS.

Kinetic Models	Parameters	DD@MOF/D-Pen	DD@MOF/DHQ
Zero order kinetic	K_0 (h^{-1})	0.282	0.592
model	M_0	38.844	27.151
	R^2	0.154	0.482

First order kinetic model	K_1 (h^{-1})	1.893	0.450
	$LogM_0$	53.319	60.758
	R^2	**0.993**	**0.983**
Higuchi kinetic model	K_H ($h^{-1/2}$)	3.344	6.088
	M_0	34.113	19.704
	R^2	0.285	0.668
Korsmeyer-Peppas kinetic model	N	0.094	0.205
	K_{kp}(h^{-n})	40.841	28.294
	R^2	0.726	0.857

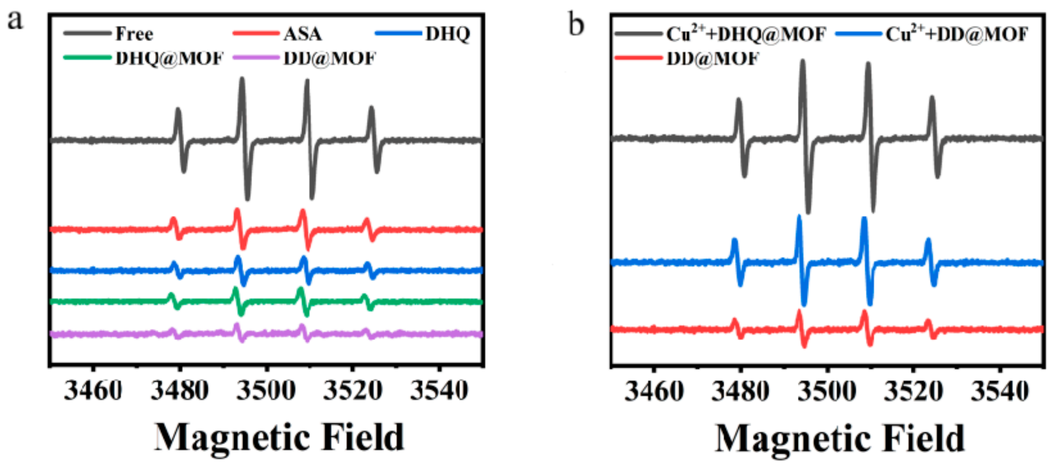

Figure S13. Determination of antioxidant capacity of different concentrations of ASA, DHQ, DHQ@MOF, DD@MOF, Cu^{2+}+DHQ@MOF and Cu^{2+}+DD@MOF by EPR.

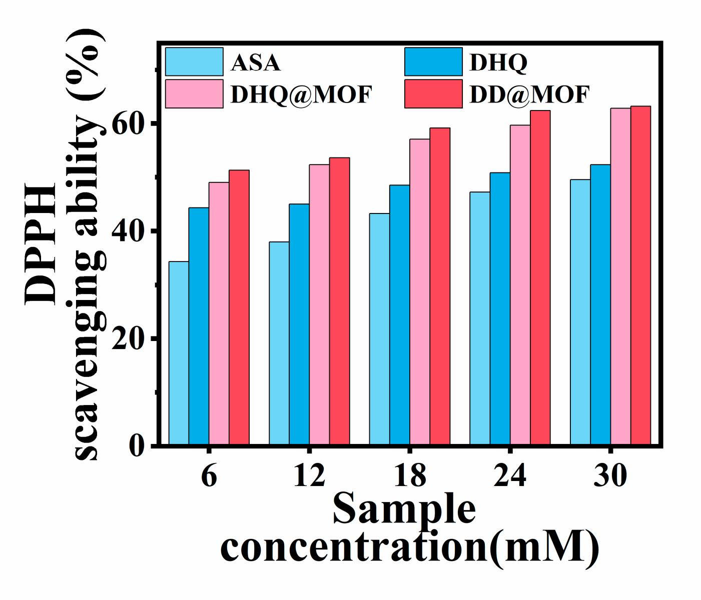

Figure S14. Determination of antioxidant capacity of different concentrations of ASA, DHQ, DHQ@MOF and DD@MOF by DPPH method.

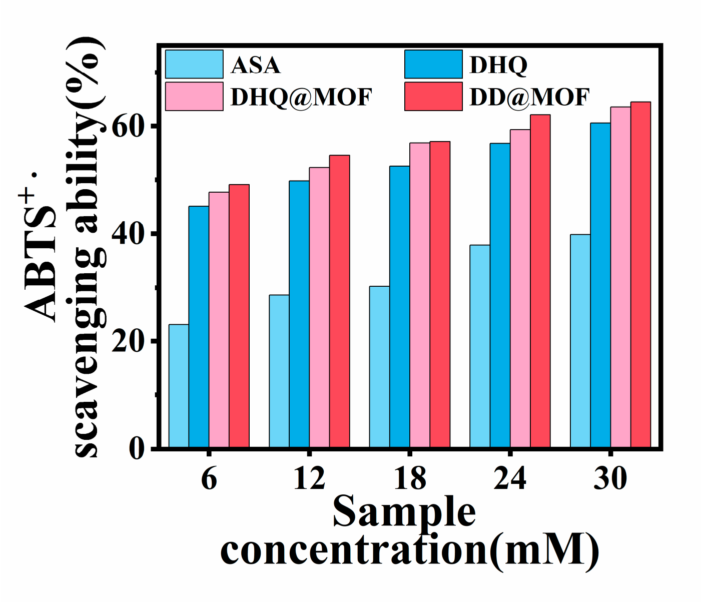

Figure S15. Determination of antioxidant capacity of different concentrations of ASA, DHQ, DHQ@MOF and DD@MOF by ABTS method.

Table S3. Comparison of the half-inhibition concentration (IC_{50}) of MOF in ABTS and DPPH free radical scavenging experiments under different conditions

	ABTS			DPPH		
	Cu^{2+}+DHQ @MOF	Cu^{2+}+DD @MOF	DD@MOF	Cu^{2+}+DHQ @MOF	Cu^{2+}+DD@ MOF	DD@MOF
R^2	0.974	0.995	0.991	0.976	0.991	0.986
IC_{50} (μg/ml)	42.733	13.320	8.711	33.510	7.415	6.953

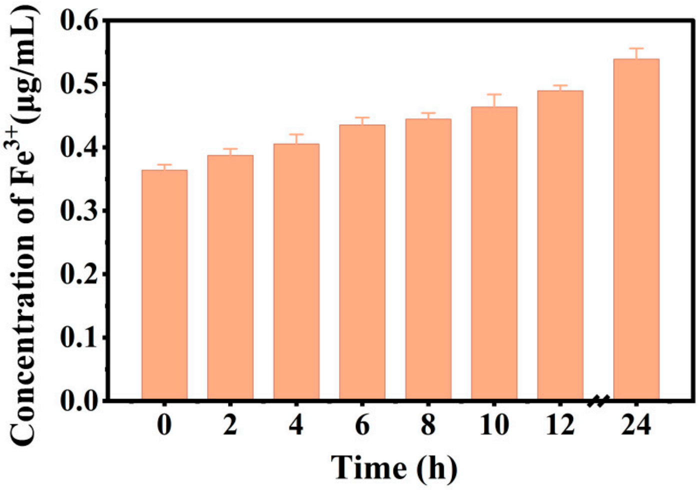

Figure S16. Time-dependent accumulation of Fe^{3+} in mouse brains after DD@MOF administration.

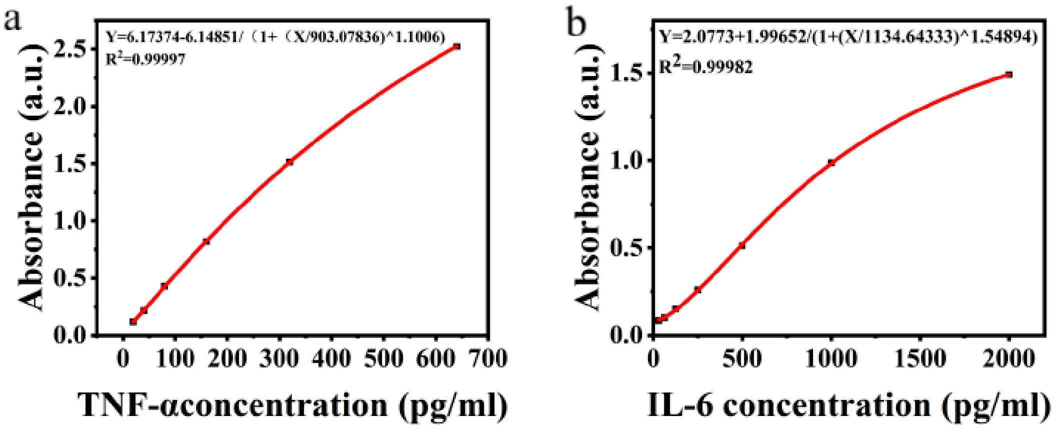

Figure S17. Standardized curves for TNF-α and IL-6 levels.

.

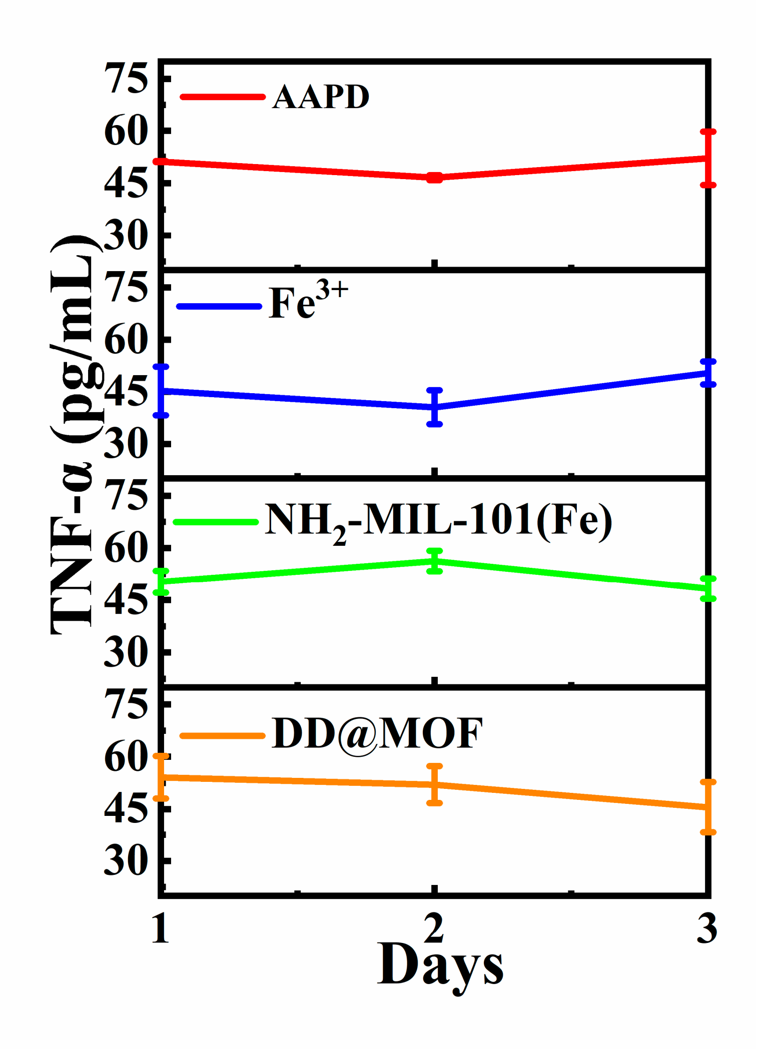

Figure S18. Images of TNF-α levels in each group of the biosafety experiment.

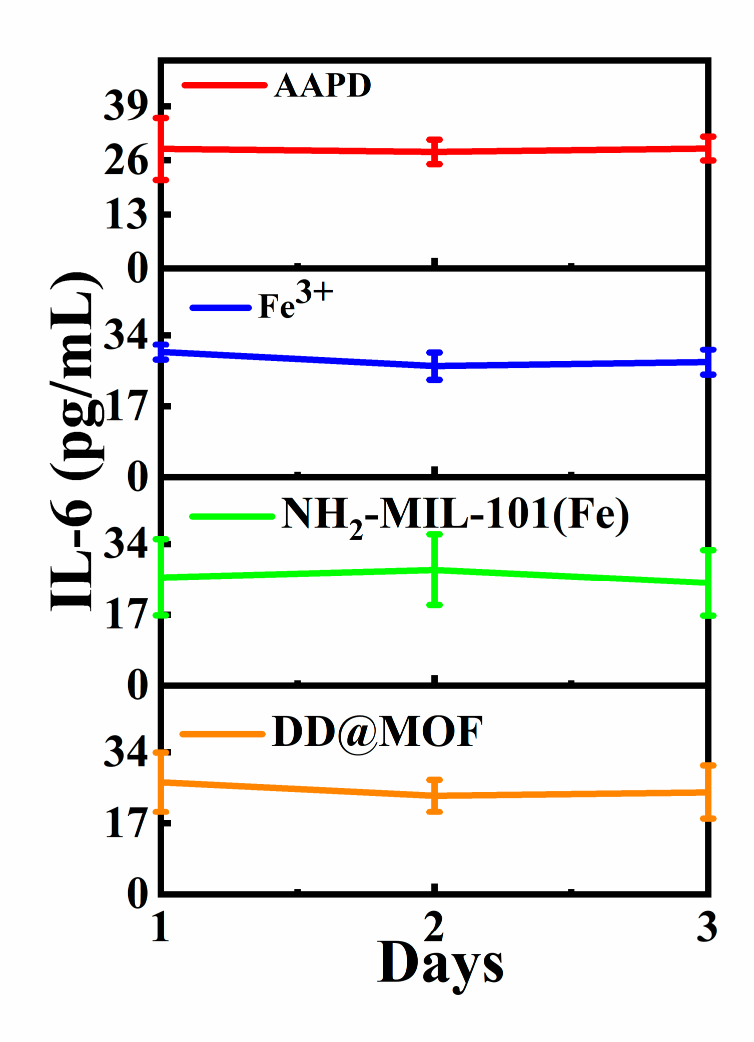

Figure S19. Images of IL-6 levels in each group of the biosafety experiment.

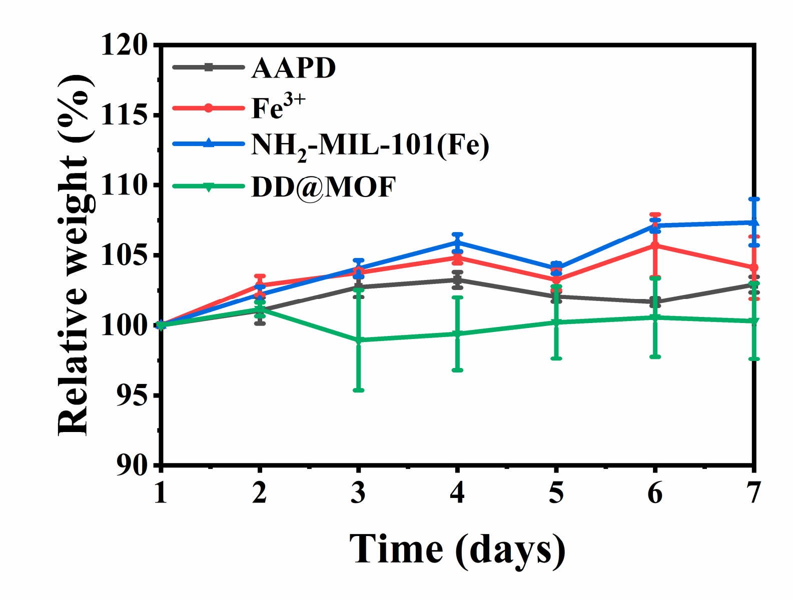

Figure S20. Images of body weight changes in each group of the biosafety experiment.

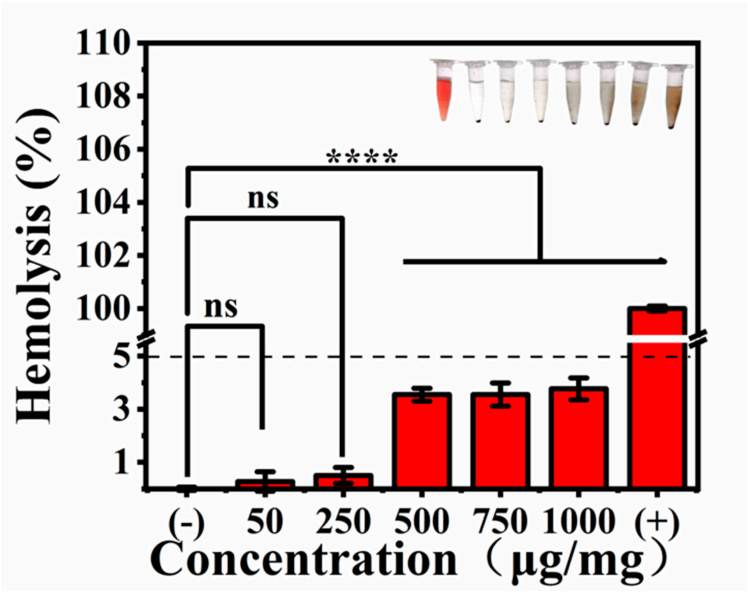

Figure S21. Hemolysis test for the biosafety experiment

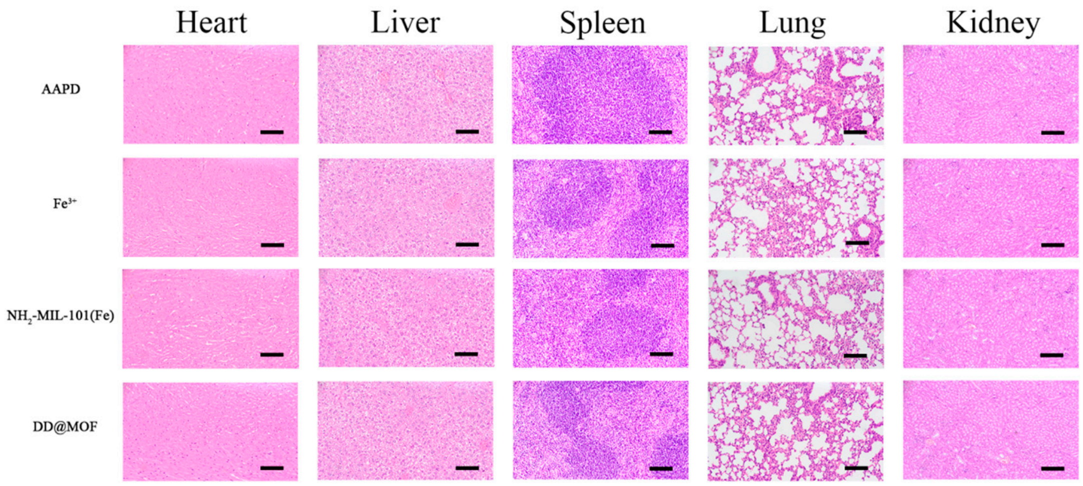

Figure S22. H&E staining of major organs for biosafety experiments (scale bars are 200 μm).

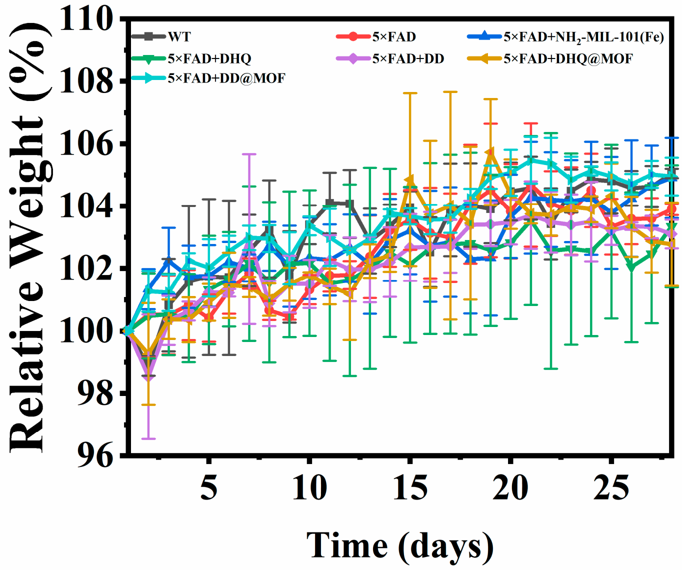

Figure S23. Changes in body weight of mice during 4 weeks.

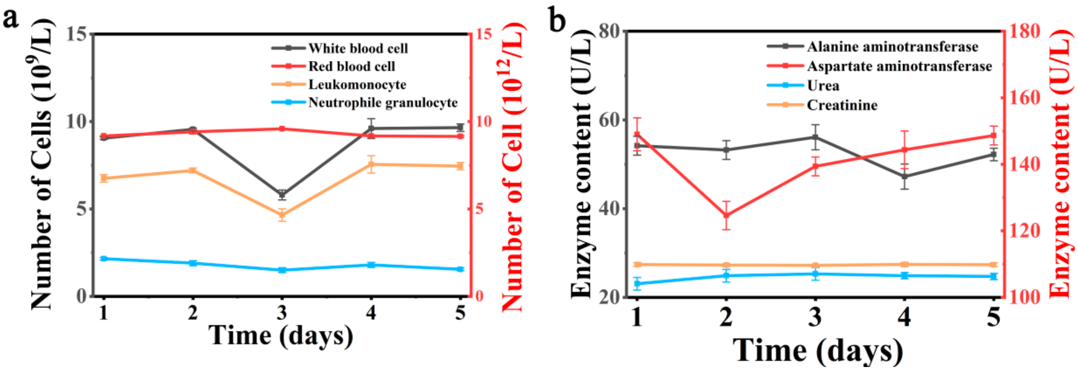

Figure S24. Trends in Hematological and Biochemical Parameters in Mice

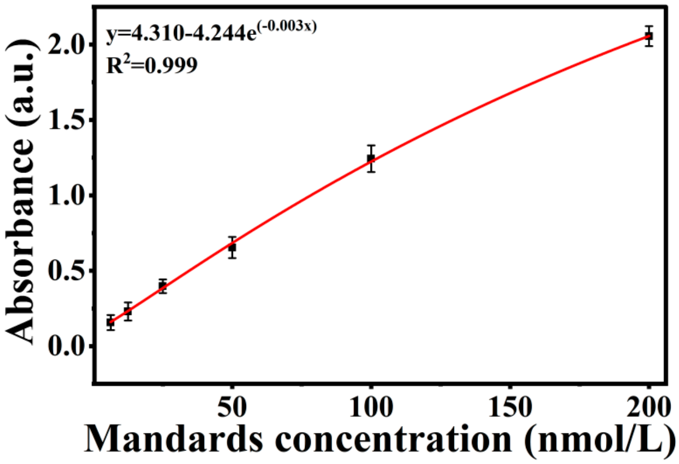

Figure S25. Standardized curves for AChE.

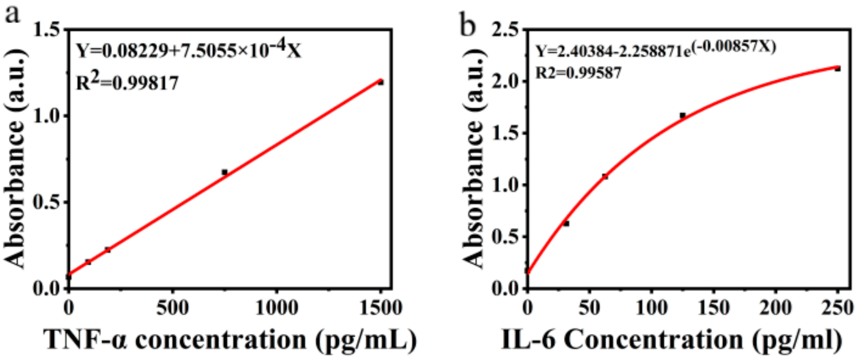

Figure S26. Standardized curves for TNF-α and IL-6 levels

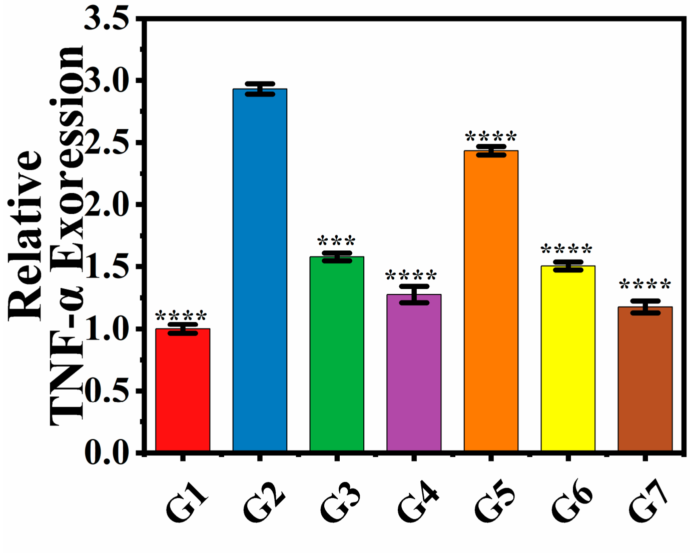

Figure S27. the expression of TNF-α in the brain of the mice. (G1: WT; G2: 5 × FAD; G3: 5 × FAD + MOF; G4: 5 × FAD + DHQ; G5: 5 × FAD + DD; G6: 5 × FAD + DHQ@MOF; G7: 5 × FAD + DD@MOF)

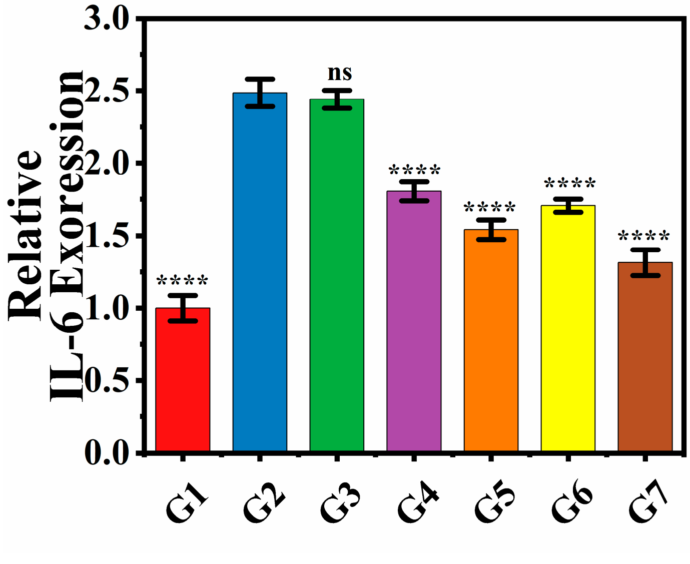

Figure S28. the expression of IL-6 in the brain of the mice. (G1: WT; G2: 5 × FAD; G3: 5 × FAD + MOF; G4: 5 × FAD + DHQ; G5: 5 × FAD + DD; G6: 5 × FAD + DHQ@MOF; G7: 5 × FAD + DD@MOF)

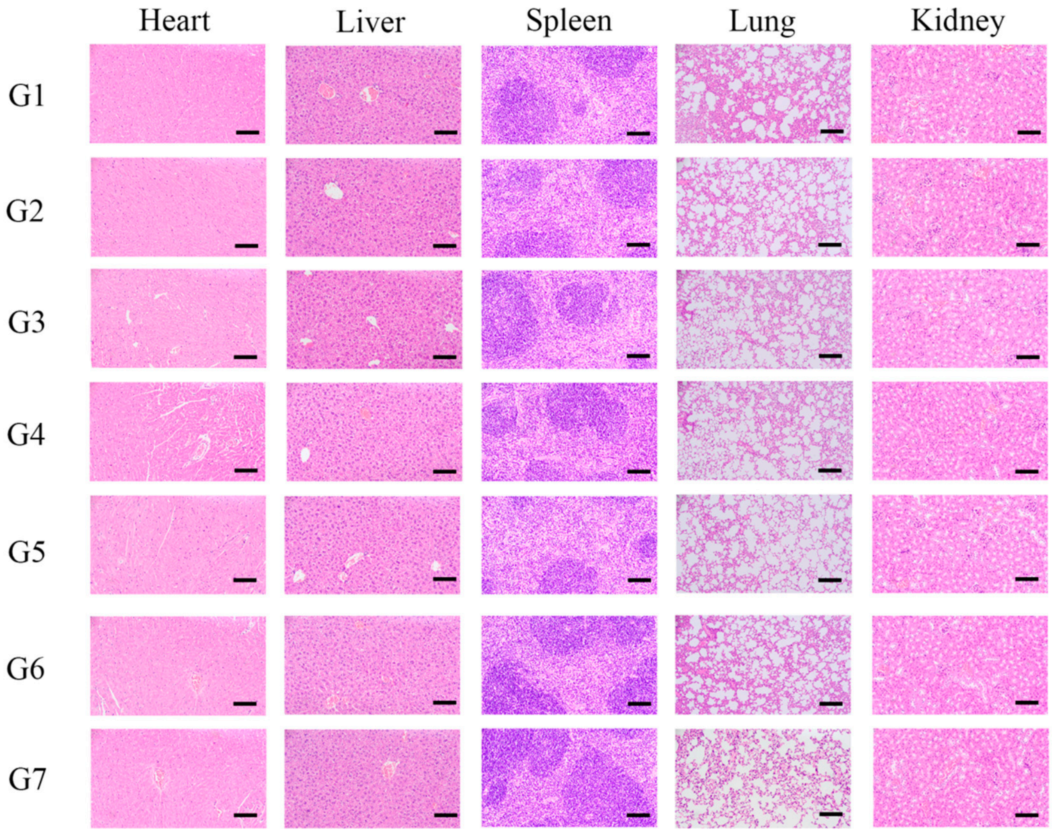

Figure S29. H&E staining of major organs for biosafety experiments (scale bar s are 200 μm). (G1: WT; G2: 5 × FAD; G3: 5 × FAD + MOF; G4: 5 × FA D + DHQ; G5: 5 × FAD + DD; G6: 5 × FAD + DHQ@MOF; G7: 5 × FA D + DD@MOF)

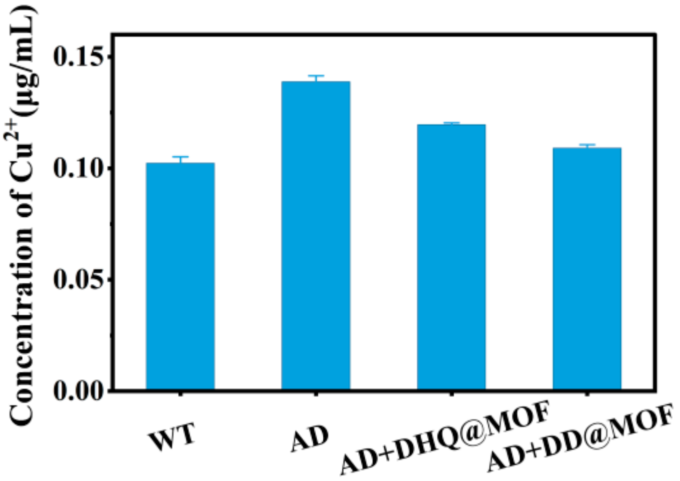

Figure S30. Copper ion content in mouse brains under different treatments.
